# Family dynamics on mental health: a network analysis

**DOI:** 10.1038/s44184-025-00168-0

**Published:** 2025-10-23

**Authors:** Zixin Jiang, Irena Tetkovic, Sharon Neufeld, Tamsin Ford, Bin-Bin Chen

**Affiliations:** 1 Department of Psychology, Fudan University, Shanghai, China; 2 Department of Psychiatry, University of Cambridge, Cambridge, UK

**Keywords:** Psychology, Signs and symptoms

## Abstract

The dynamic relationships between children’s, parents’, and siblings’ mental health have yet to be systematically explored. The present study employed network analysis to investigate concurrent and longitudinal associations of mental health symptoms in children during early childhood and their parents and siblings. A total of 3750 cohort members (47.9% female; Mage = 3.1 at T1), along with their mothers, fathers, and older siblings from the Millennium Cohort Study (MCS), were assessed for mental health problems at two waves spaced two years apart. Contemporaneous networks revealed extensive associations between intra-individual and inter-individual symptoms within the family. The older siblings’ symptoms were the strongest bridging symptoms connecting to their younger siblings. Temporal networks demonstrated directional effects from parent to child, father to mother, and older sibling to younger sibling. Maternal depressive feelings exhibited the strongest predictive effect in the family network. Overall, our findings suggest the spillover effect of mental health problems within families, underscoring the need to consider the psychological symptoms of other family members when treating individual symptoms.

## Introduction

Mental health is a significant public health issue, with one in two people worldwide experiencing a mental health disorder in their lifetime^1,2^. Within this context, the family plays a critical role for individual members’ mental health, as they must continuously adjust to stressors and changes faced both individually and collectively, as outlined in Minuchin’s family systems theory^3^. Bowen’s family system theory also emphasizes that individual symptoms, whether physical, emotional, or social, are better understood as manifestations of intense emotional process in the family rather than as individual psychopathology^4^. Spillover theory further suggests that dynamics within one family subsystem, such as the interparental relationship, can affect others, like the sibling relationship^5,6^. Consequently, when one family member experiences poor mental health, the entire family system is often impacted^7^. Given that the family serves as a crucial resource for individual psychological development in early life, understanding transactional dynamics of mental health among young children, their parents, and older siblings is essential for informing the development of more targeted and effective interventions^6,8^.

Family mental health research has provided increasing evidence that family members bidirectionally influence each other’s mental health. Parents constitute the initial subsystem in the family system. The reciprocal influence of mental health between parents represents a significant phenomenon, which can now be studied at the symptom level with a more granular approach, using network approaches. A recent study using a network approach demonstrated that patterns in symptoms were similar for both parents after having a baby, with difficulty in one area often mirrored in the other parent^9^. Symptoms such as insomnia, sadness, anhedonia, overwhelm, self-harm ideation, and guilt in one parent were associated with high levels of these symptoms in the other parent. Mental health similarity between parents has been shown to potentially arise from non-random (assortative) mating^10^. Individuals with mental health problems tend to select partners who exhibit similar psychiatric traits^11^. Such couples may subsequently influence each other’s mental health through ongoing interpersonal dynamics.

Parental mental health influences children and, in turn, parental mental health is influenced by their children. Extensive evidence has shown bidirectional influences between parents and children, and recent work has begun to use network analytic approaches to explore these dynamics at the symptom level in greater detail. For example, Chen et al. (2025)^12^ found that in early adolescence, mothers’ depressive symptoms prospectively predicted their children’s depressive symptoms^12^. As the children progressed into mid to late adolescence, a bidirectional relationship emerged between fathers and children. Interestingly, mothers’ depressive symptoms were more likely to be jointly influenced by both fathers and children. Zhang et al. (2025) similarly found a transmission effect of depression within the bidirectional dynamics of the parent-child and interparental relationships^8^. These findings underscore the complexity of interactions between parents and children, emphasizing the need for comprehensive approaches that consider interparental and parent-child dynamics in both research and interventions.

Similarly, sibling relationships play an underexplored yet crucial role in family mental health. Individuals with siblings who have mental health problems are at greater risk of poorer mental health, influenced by multifaceted factors spanning genetic, environmental, and family dynamics^13,14^. Social learning theory provides a framework for understanding sibling influence, as siblings may model behaviours and strategies observed in one another due to their close age and shared environment^15^. However, existing evidence on reciprocal mental health effects between siblings remains mixed. For instance, studies using the Strengths and Difficulties Questionnaire (SDQ) have shown that older siblings’ internalizing and externalizing problems, as well as their prosocial behaviour, predict similar behaviour in younger siblings, while effects in the reverse direction are less consistent^16,17^. Yet, existing sibling studies fail to fully account for parental influences, overlooking the broader family context^18^.

Whilst the literature acknowledges the complexity of the family and suggests associations between parents’, children’s, and siblings’ mental health, gaps remain in understanding the spillover effects of mental health symptoms among fathers, mothers, and both older and younger children in multi-child families. More specifically, it is unclear which family members’ symptoms influence family members the most. Addressing these gaps might help clinicians understand, target, and better assist these families^19^.

To address these limitations, this study utilized a network approach to explore the dynamic relationships of mental health symptoms within families. Since psychopathology manifests at the symptom level, the network approach provides a more granular means of capturing differential changes over time in symptoms. This approach can also help distinguish central symptoms from key connecting (bridge) symptoms, thereby providing even greater detail about how symptoms react within and across family members. We analyzed family mental health data from the UK Millennium Cohort Study (MCS) when the cohort members were aged three and five. Analyses include the cohort members (younger siblings in this study), their older siblings, and their parents. All mental health symptoms were reported by parents, covering both their own symptoms and those of their children.

The objectives of this exploratory study are as follows: we first constructed two contemporaneous networks to capture granular symptom-level interactions within and across family members, using two waves of data two years apart. We aimed to explore whether and how symptoms co-occur within and between family members. We then calculated network centrality indices to identify which family member’s symptoms would be central in the network and which symptoms would connect to the mental health of different family members. Finally, we built a cross-lagged network to assess the stability of symptoms and whether the symptoms of one individual could predict those of others over time.

## Methods

### Participants

Data were extracted from the Millennium Cohort Study (MCS) project across two waves of data collection: Wave 2 and Wave 3. The MCS project followed 18,552 children (cohort members) and their families born in England, Scotland, Wales, and Northern Ireland between 2000 and 2002. Data in this study were collected from cohort members (referred to as younger siblings in the present study), mothers, fathers, and older siblings at waves 2 and 3. Cohort members were excluded if family member data were not complete. In cases where a cohort member has two or more siblings, data from the older sibling closest in age to the cohort member were included, and we adjusted for the number of siblings in the network analyses. To control for the effect of kinship, we only included families where the cohort member was biologically related to parents and siblings. A total of 3,750 families, all of whom participated in both wave 2 and wave 3, remained for analyses (see Supplemental Materials Fig. S1 for a flow chart showing the selection of analytic samples). At wave 2, the average ages of younger siblings were 3.1(*SD* = 0.2), while older siblings were 6.7 (*SD* = 2.2), mothers were 34.7 (*SD* = 4.8), and fathers were 37.2 (*SD* = 5.4) years. The average time gap between wave 2 and wave 3 assessments was 2.1 years (SD = 0.3). Just over half (52.1%) of older siblings were girls (*n* = 1955), and 47.9% of younger siblings were girls (*n* = 1795). Most families were from England (63.1%), with representation across the United Kingdom (Wales 15%, Scotland 11.4%, and Northern Ireland 10.5%). The demographic characteristics of the study sample and the MCS full sample at wave 2 and wave 3 are presented in Supplemental Materials Table S1.

### Measures

A parental respondent, who was the mother in approximately 99% of cases, reported on both their children using the Strengths and Difficulties Questionnaire (SDQ) at waves 2 and 3^20^. The SDQ contains five subscales with five items each, measuring emotional symptoms, conduct problems, peer problems, and prosociality. Responses are on a 3-point Likert scale, with 0 meaning “not true”, 1 meaning “somewhat true”, and 2 meaning “certainly true”, with higher scores indicating greater difficulties except for the prosocial scale, where they indicate better function. In line with prior studies assessing symptom networks across multiple domains, mean subscale scores were used in our analysis^21^. The items within each subscale are highly similar and overlapping (see Supplemental Materials Table S2 for the Cronbach’s alpha coefficients), and including all items as separate nodes in the network could obscure cross-domain symptom associations both within and between individuals. In addition, reducing the number of redundant items also improved the statistical power of the network analysis.

Parental psychological distress was measured with the six-item version of the Kessler Psychological Distress Scale (K6), which is a brief, reliable, and valid tool used to identify mental health disorders in adults^22,23^. The K6 assesses the frequency with which individuals felt depressed, hopeless, restless/fidgety, that everything was an effort, worthless, and nervous. Mothers and fathers rated their feelings over the past 30 days on a 5-point Likert scale [1 = All of the time, 2 = Most of the time, 3 = Some of the time, 4 = A little of the time, 5 = None of the time]. The K6 is scored by reverse-scoring the items so higher scores indicate greater distress.

### Data preparation

All analyses were conducted in R Version 4.3.1 and R*Studio* Version 2023.06.2 + 561^24^. Among our analytic sample, 2,363(63%) cases had missing values across wave 2 and wave 3. Assuming that missing values were missing at random (MAR), multiple imputation was conducted using the mice R package^25^. To strengthen the plausibility of the MAR assumption, our imputation model included not only mental health variables but also a set of auxiliary variables, such as social stratum, age spacing between siblings, sibling sex constellation, parental education, and the number of children in the family. According to White’s recommendation, the number of imputations should be at least equal to the percentage of incomplete cases^26^. Thus, data were aggregated using Rubin’s rules across 65 multiply imputed datasets, as 63% of cases were missing. Outliers were not found in the dataset (see Supplemental Materials Table S3).

### Network estimates

Network analyses were performed with the *bootnet* (1000 iterations used)^27^, *glmnet* (regularized regression models)^28^, *networktools* (bridge nodes estimation)^29^, and *qgraph* (network visualization)^30^ R packages. The nodes included in the network were all items of the K6 for each mother and father, and all SDQ subscales for each older and younger sibling. Some demographic variables may affect the mental health of family members in multiple-child families^27^. Thus, the networks also included the following variables as covariates: age spacing between siblings (also to adjust for older siblings’ age, as the younger sibling’s age was nearly fixed), sex constellation of siblings (the same or mixed sex), education status of mothers and fathers (UK’s National Vocational Qualification (NVQ) level), family socioeconomic status (advantaged and disadvantaged areas, or areas with higher ethnic minority density, based on area-level socioeconomic stratification in the MCS)^28^, and the number of children in the family. All continuous covariates were standardized, and categorical covariates were coded as dummy variables.

First, a mixed graphical model (MGM) was estimated at T1 and T2, considering both continuous nodes (e.g., SDQ subscales and K6 items) and categorical nodes (e.g., sex constellation). The network consists of nodes and edges (representing the correlations between pairs of nodes after adjusting for the influence of all other nodes)^27^. The least absolute shrinkage and selection operator (LASSO) statistical regularization algorithm with 10-fold cross-validation was also used to remove false-positive edges by reducing weak or spurious edges to zero. Additionally, the extended Bayesian information criterion (EBIC) was employed to select the best fitting model with the lowest EBIC value out of 100 estimated^31^. Secondly, cross-lagged panel network (CLPN) analyses were employed to estimate the auto-regressive coefficient (where a node at time T1 predicts itself at time T2) and the cross-lagged coefficient (where a node at time T1 predicts other nodes at time T2 after accounting for all other nodes at time T1) through a series of node regressions^32^.

### Centrality estimates

Centrality indices were estimated to assess the relative importance of a node in relation to other nodes in the network. For the contemporaneous networks, expected influence (EI) was calculated by aggregating the raw values of the edge weights connected to that node^33^. Nodes with higher EI exhibit stronger connections with others in the whole network. Among various centrality indices, EI is considered more robust, as it takes into account only the direct connections between nodes^34^. The centrality bootstrapped difference test assessed whether the node with the highest or lowest EI was significantly different from other nodes. To identify which symptoms may transmit across family members, the bridge expected influence (EI) was also computed. Bridge EI illustrates the connection of a node from one cluster (e.g., a mother symptom) to the sum of all the connections to nodes in another cluster (e.g., younger child symptoms). In the present study, each cluster corresponds to a different family member, allowing bridge EI to capture potential connections between symptoms across family members. Bridge EI bootstrapped difference tests were conducted to show whether nodes with the highest bridge EI were significantly different from other nodes.

For the temporal network, cross-lagged out-expected influence (out-EI) and in-expected influence (in-EI) were calculated. Out-EI refers to the degree to which each node predicts other nodes at the next wave in the network (the sum of all outgoing edges connected to a node), and in-EI indicates the degree to which each node is predicted by other nodes at the prior wave in the network (the sum of all incoming edges connected to a node). Further, predictability was calculated from the temporal network to indicate how well a node at T2 could be predicted by all the T1 nodes it was connected to in the network (i.e., amount of variance explained)^28^.

### Network stability and accuracy

The accuracy and stability of network metrics were estimated using two bootstrapped estimation procedures^27^. To assess the accuracy of edge weights, edge weights with a 95% confidence interval (CI) were computed using nonparametric bootstrapping. Edge-weight bootstrapped difference tests were conducted to determine whether an edge was significantly stronger than other edges. Edge differences were considered significant if the 95% bootstrapped confidence interval did not include zero^27^. For the stability of centrality metrics (calculated for EI, bridge EI, out-EI, and in-EI), correlation stability (CS) was assessed using case-drop bootstrapping. Coefficient values of CS ≥ 0.25 were deemed acceptable, and values ≥ 0.50 were considered indicative of good stability^27^. Furthermore, we conducted a Network Comparison Test (NCT) to assess differences in the structure of contemporaneous networks across time points^12^.

### Ethical considerations

The MCS Study was approved by London Multi Centre Research Ethics Committees. Referenced as MREC/03/2/022 for wave 2 and 05/MRE02/46 for wave 3. Written informed consent was obtained from parents or guardians of cohort children, as well as from other participants when required. The present analyses used anonymised secondary data from the UK Data Service, and no further ethical approval or consent was required.

## Results

### Contemporaneous networks

**Fig. 1:**
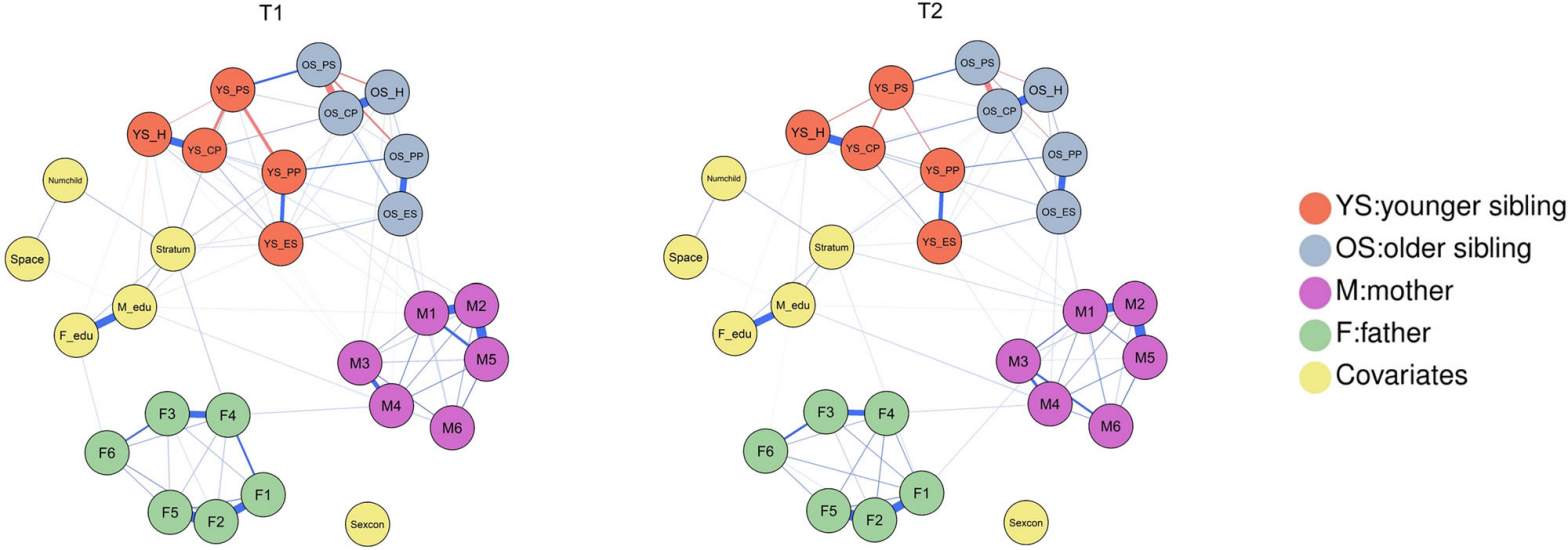
Contemporaneous networks. ES = emotion symptom; CP = conduct problem; H = hyperactivity; PP = peer problem; PS = pro-social; M1 = mother felt depressed; M2 = mother felt hopeless; M3 = mother felt restless/fidgety; M4 = mother felt everything an effort; M5 = mother felt worthless; M6 = mother felt nervous; F1 = father felt depressed; F2 = father felt hopeless; F3 = father felt restless/fidgety; F4 = father felt everything an effort; F5 = father felt worthless; F6 = father felt nervous; Stratum = social stratum; Space = age spacing between siblings; Sexcon = sex constellation of siblings; M_edu = educational level of mother; F_edu = educational level of father; Numchild = number of child within the family; T1 = time 1; T2 = time 2. Nodes represent mental health symptoms of family members and covariates. Edges represent undirected associations between nodes at the same time point. Blue edges indicate positive associations, whereas red edges indicate negative associations. Edge thickness and boldness reflect the strength of associations.

The contemporaneous networks of mothers, fathers, and older and younger siblings within the family during T1 and T2 are shown in Fig. 1. The undirected edges of inter-individual and intra-individual nodes are presented in the Supplementary Table S4. The intra-individual nodes were interconnected. The strongest undirected edges at both T1 and T2 were hopelessness and worthlessness for fathers (F2-F5, *r* = 0.41) and mothers (M2-M5, *r* = 0.40), and conduct problem and hyperactivity for older siblings (OS_CP-OS_H, *r* = 0.35) and younger siblings (YS_CP-YS_H, *r* = 0.33). The inter-individual nodes within the family were also interconnected. The strongest inter-individual connection at both T1 and T2 was the association between prosociality in younger siblings and those in older siblings (YS_PS-OS_PS, *r* = 0.17). Edge-weight bootstrapped difference tests indicated that the strongest edges mentioned above did not differ significantly from each other but were significantly stronger than the weaker edges ranked below them at both T1 and T2 (see Supplemental Materials, Figs. S2–S3). The network comparison test showed that the overall network structure differed significantly between T1 and T2 (M = 0.246, *p* < 0.001). Although most edges did not differ significantly between T1 and T2, the overall structure of the family network changed over time (see Table S5 for details).

**Fig. 2:**
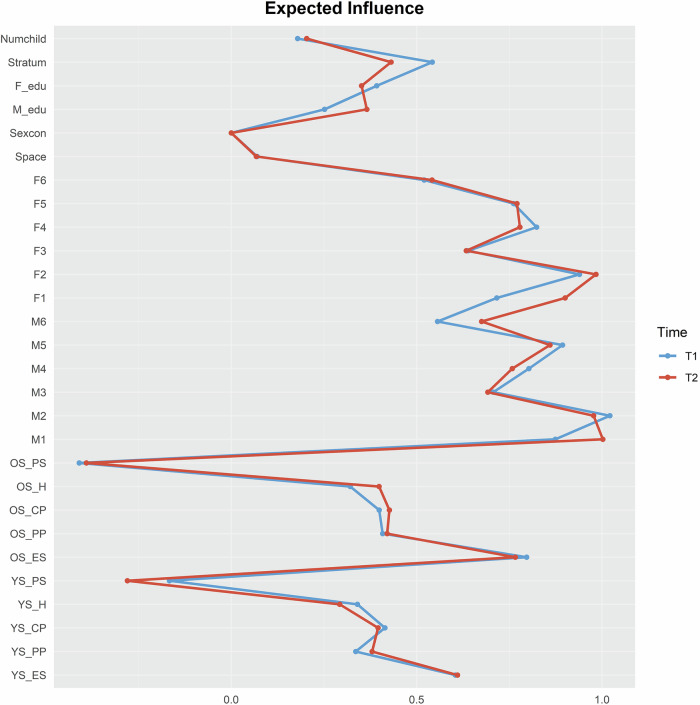
Expected influence (EI) of contemporaneous networks. YS = younger sibling; OS = older sibling; ES = emotion symptom; CP = conduct problem; H = hyperactivity; PP = peer problem; PS = pro-social; M1 = mother felt depressed; M2 = mother felt hopeless; M3 = mother felt restless/fidgety; M4 = mother felt everything an effort; M5 = mother felt worthless; M6 = mother felt nervous; F1 = father felt depressed; F2 = father felt hopeless; F3 = father felt restless/fidgety; F4 = father felt everything an effort; F5 = father felt worthless; F6 = father felt nervous; Stratum = social stratum; Space = age spacing between siblings; Sexcon = sex constellation of siblings; M_edu = educational level of mother; F_edu = educational level of father; Numchild = number of child within the family. EI indicates the sum of the connections of a given node with other nodes.

**Fig. 3:**
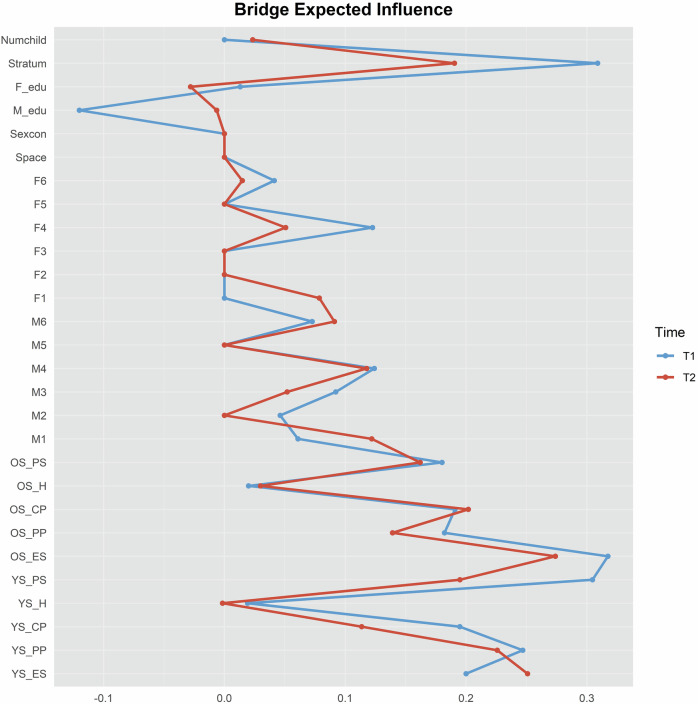
Bridge EI of contemporaneous networks. YS = younger sibling; OS = older sibling; ES = emotion symptom; CP = conduct problem; H = hyperactivity; PP = peer problem; PS = pro-social; M1 = mother felt depressed; M2 = mother felt hopeless; M3 = mother felt restless/fidgety; M4 = mother felt everything an effort; M5 = mother felt worthless; M6 = mother felt nervous; F1 = father felt depressed; F2 = father felt hopeless; F3 = father felt restless/fidgety; F4 = father felt everything an effort; F5 = father felt worthless; F6 = father felt nervous; Space = age spacing between siblings; Sexcon = sex constellation of siblings; M_edu = educational level of mother; F_edu = educational level of father; Numchild = number of child within the family. Bridge EI indicates the extent to which a node connects across different clusters (family members).

Regarding the centrality of the symptoms in the network, EI was estimated to quantify the sum of the connections of a given node with other nodes. The symptoms with the highest EI were hopeless feelings in mothers (M2) at T1, and depressive feelings in mothers (M1) at T2 (see Fig. 2). The symptoms with the lowest EI were prosociality in older sibling (OS_PS) at T1 and T2. Additionally, the sex constellation of siblings (Sexcon) as a covariate exhibited the lowest EI at both time points. Bootstrapped difference tests for centrality showed that the node with the highest or lowest EI was significantly different from most other nodes (see Supplemental Materials Figs. S4–S5). In addition, bridge EI was calculated to assess the extent to which a node connects across different family members. Child nodes had the highest bridge EI connected to the same nodes of their siblings (see Fig. 3). The highest bridge EIs were: at T1, emotional symptoms in older siblings (OS_ES, bridge EI = 0.32) and prosociality in younger siblings (YS_PS, bridge EI = 0.30); at T2, emotional symptoms in both older siblings (OS_ES, bridge EI = 0.27) and younger sibling (YS_ES, bridge EI = 0.25). Parents’ nodes showed the highest bridge EI to the same nodes of their partners: at T1, the feeling that everything was an effort in both mothers (M4, bridge EI = 0.12) and fathers (F4, bridge EI = 0.12); at T2, depressive feelings in both mothers (M1, bridge EI = 0.12) and fathers (F1, bridge EI = 0.08). Bridge EI bootstrapped difference tests showed that the node with high bridge EI exhibited no significant differences from its adjacent-ranked nodes (see Supplemental Materials Figs. S6–S7).

Contemporaneous network metrics showed high stability for EI (T1 and T2: CS = 0.75, 95% CI [0.67, 1.00]) and bridge EI (T1 and T2: CS = 0.75, 95% CI [0.67, 1.00]) (see Supplemental Materials Figs. S8–S11). Bootstrapping confidence intervals (CIs) around edge weights were relatively small to moderate (see Supplemental Materials Figs. S12–S13), which suggested that the stability of the network was acceptable^27^.

### Temporal networks

**Fig. 4:**
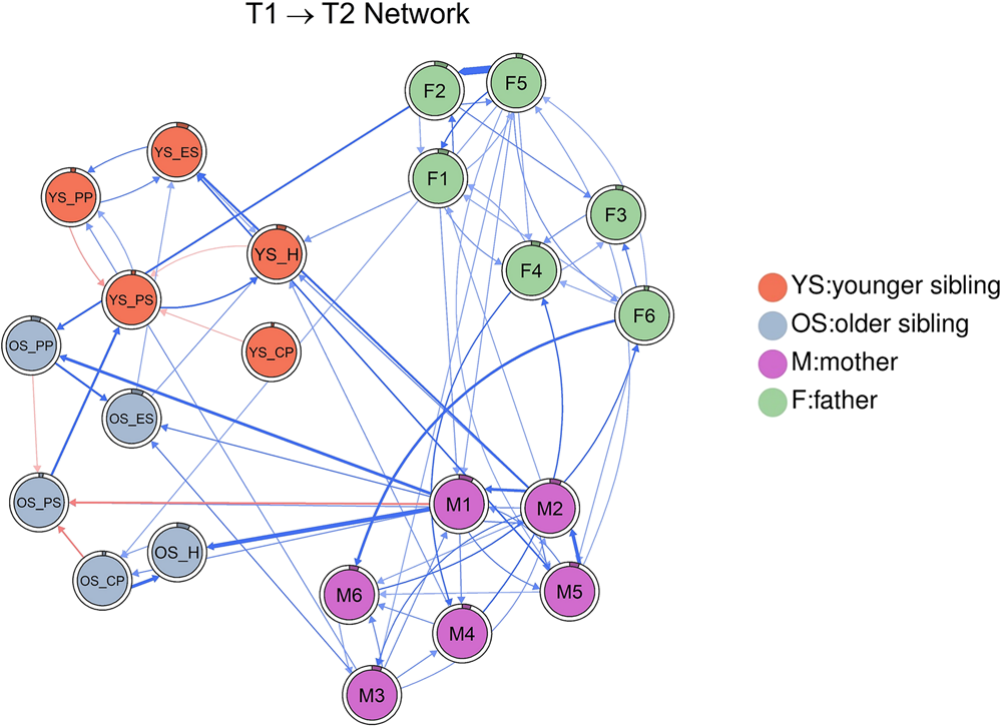
Temporal network. ES = emotion symptom; CP = conduct problem; H = hyperactivity; PP = peer problem; PS = pro-social; M1 = mother felt depressed; M2 = mother felt hopeless; M3 = mother felt restless/fidgety; M4 = mother felt everything an effort; M5 = mother felt worthless; M6 = mother felt nervous; F1 = father felt depressed; F2 = father felt hopeless; F3 = father felt restless/fidgety; F4 = father felt everything an effort; F5 = father felt worthless; F6 = father felt nervous; T1 = time 1; T2 = time 2. Nodes represent mental health symptoms of family members. Covariates were omitted in the graph. Edges represent cross-lagged effects, where a node at time T1 predicts other nodes at time T2. Blue edges indicate positive associations, whereas red edges indicate negative associations. Edge thickness and boldness reflect the strength of associations. The pie chart around a node indicates the predictability of that node in the network. Predictability represents the proportion of variance in each T2 node explained by its connected T1 node.

The temporal network is shown in Fig. 4. Edges within and across clusters were estimated by CLPN analysis (see Supplemental Materials Table S6). For autoregressive edges, the highest scoring was hyperactivity in older siblings (OS_H, *β* = 0.59), emotion symptoms in younger siblings (YS_ES, *β* = 0.36), restlessness/fidgetiness in fathers (F3, *β* = 0.33), and the feeling that everything is an effort in mothers (F4, *β* = 0.28). The autoregressive edges (mean *β* = 0.29) were generally higher than the cross-lagged edges (mean *β* = 0.02). After regularization convergence and omission of autoregressive edges in the network, there were 317 non-zero cross-lagged edges. We excluded autoregressive edges, zero, and weak cross-lagged edges from the graph to improve visualization. The greatest cross-lagged edges between intra-individual nodes were from worthlessness to hopelessness in fathers (F5 → F2, *β* = 0.17) and mothers (M5 → M2, *β* = 0.11), from hopelessness to depressive feelings in mothers (M2 → M1, *β* = 0.11), and from conduct problem to hyperactivity in older siblings (OS_CP → OS_H, *β* = 0.11). In terms of cross-lagged edges of inter-individual nodes, there were more cross-lagged edges from mothers’ nodes to child nodes. The strongest cross-lagged edges were from depressive feelings in mothers to hyperactivity (M1 → OS_H, *β* = 0.13) and to peer problems(M1 → OS_PP, *β* = 0.11) and to prosociality (M1 → OS_PS, *β* = −0.10) in older siblings, as well as from hopelessness in mothers to emotional symptoms in younger siblings (M2 → YS_ES, *β* = 0.10). The strongest cross-lagged edge between father and child was from hopelessness in fathers to peer problems in older siblings (F2 → OS_PP, *β* = 0.09). For cross-lagged edges between mother and father, there was an effect from nervousness in fathers to nervousness in mothers (F6 → M6, *β* = 0.10), as well as reciprocal effects for ‘everything was an effort,’ with both edges showing the same effect (M4 → F4, *β* = 0.09; F4 → M4, *β* = 0.09). For cross-lagged edges between siblings, the strongest effect was from prosociality in older siblings to prosociality in younger siblings (OS_PS → YS_PS, *β* = 0.10). Edge-weight bootstrapped difference tests indicated that each strongest edge above was significantly stronger than the subsequent strongest edge (see Supplemental Materials Figs. S14).

**Fig. 5:**
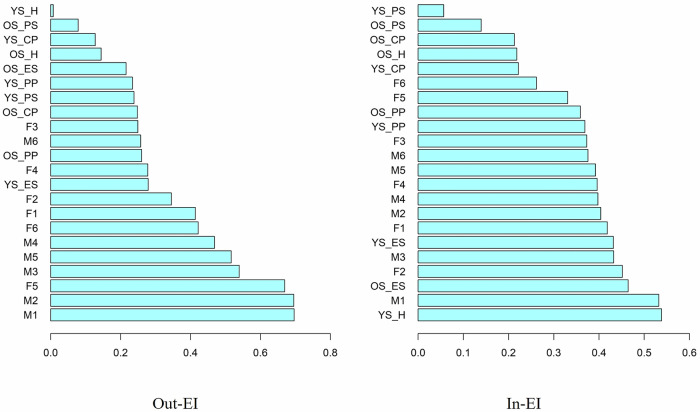
Out-EI and in-EI of temporal network. YS = younger sibling; OS = older sibling; ES = emotion symptom; CP = conduct problem; H = hyperactivity; PP = peer problem; PS = pro-social; M1 = mother felt depressed; M2 = mother felt hopeless; M3 = mother felt restless/fidgety; M4 = mother felt everything an effort; M5 = mother felt worthless; M6 = mother felt nervous; F1 = father felt depressed; F2 = father felt hopeless; F3 = father felt restless/fidgety; F4 = father felt everything an effort; F5 = father felt worthless; F6 = father felt nervous. Out-EI and in-EI indicate the outgoing and incoming influence of a node on other nodes.

Out-EI and in-EI, indicating a node’s outgoing and incoming influence on other nodes, are shown in Fig. 5. The highest out-EI were depressive (M1, Out-EI = 0.70) and hopeless (M2, Out-EI = 0.70) feelings in mothers, and worthless feelings in fathers (F2, Out-EI = 0.67). The above three nodes had the most outgoing connections with T2 nodes. The highest in-EI were hyperactivity in younger siblings (YS_H, in-EI = 0.54), depressive feelings in mothers (M1, in-EI = 0.53), and emotional symptoms in older siblings (OS_ES, in-EI = 0.46). The above three nodes had the most incoming connections from the other nodes at T1. Further, depressive feelings in mothers at T2 showed the highest predictability (M1, R^2^ = 0.08) by connected nodes in the T1 network. Out- and in-EI bootstrapped difference tests showed that each node was significantly different from other nodes (see Supplemental Materials Figs. S15–S16).

Cross-lagged network metrics showed high stability for in-EI and out-EI (CS = 0.67, 95% CI [0.44, 1.00]) (see Supplemental Materials Figs. S17–S18). The 95% Bootstrapping confidence intervals (CIs) around edge weights were small to moderate (see Supplemental Materials Figs. S19), which suggested that the stability of the network was acceptable.

## Discussion

Family, as a proximal factor in the broader ecological systems, plays a crucial role in directly influencing individual mental health^19^. Yet, very few studies focus on the dynamic influence of mental health between family members, including parents, children, and siblings. Here, based on UK large-scale longitudinal data, we employed network analysis to examine the unique relationship of psychological symptoms between children, their older siblings, and their parents. Overall, contemporaneous networks revealed extensive concurrent associations between intra-individual and inter-individual symptoms within the family. The directional network demonstrated that family members’ symptoms showed relative stability, with members’ symptoms predicting each other over time.

The results of the contemporaneous family networks revealed that the interconnections between intra-individual symptoms were more pronounced than those between inter-individual symptoms. The association between intra-individual symptoms was primarily observed within specific categories of psychopathology. For both parents, there was a strong link between feelings of hopelessness and worthlessness, which were co-occurring symptoms of depression^35^. In terms of both older and younger siblings, the conduct problems and hyperactivity were found to be closely related. As has been widely established, previous research combines the behavioural and hyperactivity subscales into externalizing problems^36^.

The strongest inter-individual connection in the contemporaneous network, as reported by parents, was between siblings’ prosocial behaviours. These associations suggest that higher prosocial behaviour in one sibling tends to co-occur with higher prosocial behaviour in the other. One possible explanation is that some families may exhibit higher overall levels of prosocial behaviour than others, providing role models and a supportive environment for siblings^37^. To verify this potential mechanism, future research should longitudinally assess prosocial behaviour across all family members. Moreover, emotional symptoms in older siblings were found to be the strongest bridge symptoms connected to younger siblings’ identical symptoms. These sibling symptom associations may stem from shared genetic and environmental factors, as well as frequent interpersonal interactions. It is noteworthy that all child symptoms were reported by a parent; therefore, the observed sibling associations may differ from those obtained from child self-reports or other informants.

Maternal depressive feelings had the highest centrality in the family contemporaneous network, showing numerous associations with child symptoms. Our findings are similar to previous network analyses of depressive symptoms in father–mother–child triads, which identified “feeling depressed” as the symptom with the highest centrality in the family network^24^. While gender roles have changed over time, mothers still maintain a central role in childcare^38,39^. More frequent mother–child interactions may facilitate the transmission of emotional states between mothers and children. Nevertheless, the role of maternal depressive feelings in the family network warrants careful consideration. Since mothers reported on both their own and their children’s symptoms, the observed associations may partly reflect reporting bias. Mothers with higher depressive feelings might perceive their children’s difficulties more negatively, even if the children do not exhibit such elevated symptoms. Moreover, even if maternal depressive feelings exhibit high centrality, this does not necessarily represent a core symptom in the network. This high centrality may merely reflect shared outcomes in family mental health rather than indicate a causal role in other symptoms^34^. Finally, extensive associations among other family members further underscore the complexity of the family mental health network.

The temporal network revealed that the symptoms of each family member showed a moderate level of stability over the course of two years. Our findings suggest that symptoms that remain stable within individuals were similar across family members. For example, “everything was an effort” was the most stable symptom in mothers and the second-most stable symptom in fathers. This individual-level symptom stability appears to be linked to reciprocal influences between parents, as indicated by the cross-lagged effects of “everything was an effort” being equal in magnitude between mothers and fathers. A relatively stable environment, such as family interaction styles coupled with the psychological well-being of each family member, could contribute to symptoms becoming more trait-like^40^. Thus, it is important to consider the interconnectedness of family members’ mental health and its potential long-term effects.

The temporal network found that the most temporal edges across family members were seen from mothers to children. At the symptom level, maternal depressive feelings most strongly predicted the older sibling’s increased behavioural problems, such as hyperactivity, peer problems, and decreased prosocial behaviours; maternal hopeless feelings most strongly predicted the younger sibling’s elevated emotional symptoms. Although previous studies also identified significant longitudinal associations between maternal depression and various child symptoms, the current findings indicated that maternal depressive feelings have differential effects on the symptoms of older and younger siblings^41^. Younger children’s emotional regulation is still dependent on parent–child co-regulation during the preschool years^42^. When mothers experience elevated depressive symptoms, mother–child co-regulation may be less effective, and younger children may exhibit increased emotional difficulties. For older children, social competence and prosocial behaviour are particularly salient at this stage, as they engage more extensively with peers. Maternal depressive symptoms may reduce opportunities for modelling prosocial behaviours, which in turn could be associated with children’s social competence and behavioural outcomes^43^. However, this proposed mechanism requires further research and verification. Our findings imply the need to consider the child’s developmental stage and family roles when understanding the link between maternal depressive symptoms and child-specific outcomes.

For the temporal edges between fathers and children, the most pronounced association was that paternal hopelessness predicted peer problems in the older sibling. Fathers experiencing hopelessness may have limited involvement with their children, and father-child interactions play a unique role in developing children’s social competence^44^. Father-child interactions often involve stimulating physical play, which can ignite children’s curiosity and build their confidence in the social environment. In contrast, longitudinal associations between fathers and younger siblings were fewer or less pronounced. One possible explanation is that paternal influence on children may accumulate over development and may not yet be fully expressed in early childhood. This is consistent with evidence showing that the longitudinal associations between parental mental health and children’s psychological outcomes increase as children age^45^. Another possible explanation is that paternal influence on younger children may be modulated by the roles of mother and older sibling.

Regarding the strongest predictive effect between parents, we found that fathers’ nervousness significantly predicted mothers’ nervousness over time. Previous studies have shown that anxiety and fear are not merely individual experiences but also involve interpersonal interactions, particularly within close relationships through transference of emotions^46^. Additionally, fathers’ and mothers’ symptoms of “everything was an effort” mutually influenced each other over time. This mutual influence may partly reflect assortative mating, as people tend to select partners with similar traits, including vulnerability to stress^10^. Additionally, parents are exposed to shared stressors, such as the burden of parenting young children, which may promote emotional synchrony between parents. The combination of similar traits and shared environmental stressors is likely to facilitate reciprocal influences between parents over time.

With regard to the sibling relationship, the prosocial behaviour of older siblings predicted the subsequent prosocial behaviour of younger siblings, consistent with past findings showing the dominant role of older siblings^17^. The asymmetry between siblings may arise from the age gap, which often leads younger siblings to strive to imitate the behaviours and actions of their older siblings^47^. It should be noted that Chi and colleagues found evidence similar to ours in families with two children, whereas in families with three children, younger siblings dominate prosocial behaviour^16^. Future research needs to further explore the complex influence of siblings in families with varying numbers of children.

A key strength of this research lies in the family systems perspective, considering the spillover effect between parents, children, and siblings. While providing a holistic understanding of mental health, the study also highlights the unique effect of each family member on other members of the family. Another strength is the use of network analysis, which enables a nuanced examination of the concurrent and longitudinal associations of specific symptoms among family members. Furthermore, the use of a large-scale representative cohort survey ensures greater generalizability of results.

In terms of limitations, our results may be influenced by rater bias, as children’s mental health data were primarily reported by their mothers. Compared to reports from children or teachers, parents tend to either overestimate or underestimate their children’s symptoms^48^. For instance, relative to adolescents’ self-reports, parents typically report more positive outcomes and fewer negative symptoms^49^. These informant discrepancies underscore the importance of multi-informant approaches for a more comprehensive assessment of children’s and parents’ symptoms. Secondly, we are unable to explore indirect effects, such as the symptoms of one member serving as a mediator connecting the symptoms of two other members. Future network analysis may address this methodological limitation by examining the indirect effects. Finally, as a preliminary study exploring a psychopathology network with multiple children, the network only included psychopathology symptoms and several demographic covariates. Future research could further consider the relevant genetics and environmental factors in the family psychopathology network.

In conclusion, this study indicates extensive concurrent and longitudinal associations of psychopathology symptoms among parents, children, and siblings. Maternal depressive feelings appeared to be a central symptom associated child symptoms within the family network, highlighting the potential importance of treating parents and children together. Moreover, bidirectional relationships were observed between parents’ and siblings’ symptoms. Given the exploratory nature of this study, further research is needed to confirm whether symptoms in one family member may relate to changes in others, informing the development of targeted family interventions.

## Supplementary information

Supplementary Information

## Data Availability

The datasets analyzed during the current study are available in the MCS Data repository (https://ukdataservice.ac.uk/). Researchers with approved access in accordance with the data provider’s conditions may obtain the MCS data.

## Code availability

The underlying code for this study is not publicly available but may be made available to qualified researchers on reasonable request from the corresponding author.
