# Supplementary material for: Family dynamics on mental health: a network analysis
Source: Npj Ment Health Res. 2025 Oct 23;4:54. doi: 10.1038/s44184-025-00168-0 (PMC12549815; doi:10.1038/s44184-025-00168-0)
Supplement: Supplementary file 1 — Supplementary Information [file 44184_2025_168_MOESM1_ESM.pdf]

## Supplementary Materials

**Contents:**

**Table S1.** Demographic characteristics of the samples ........ 2
**Table S2.** Cronbach's Alphas for the Subscales of the Strengths and Difficulties Questionnaire ........ 3
**Table S3.** Descriptive statistics of variables ........ 4
**Table S4.** Edge values in contemporaneous networks ........ 5
**Table S5.** Comparison of edge weights in contemporaneous networks at T1 and T2 ........ 6
**Table S6.** Edge values in temporal network ........ 7
**Figure S1.** Flow chart of participants ........ 9
**Figure S2.** Bootstrapped difference tests of edges in the contemporaneous network at T1 ........ 10
**Figure S3.** Bootstrapped difference tests of edges in the contemporaneous network at T2 ........ 11
**Figure S4.** Bootstrapped difference tests of EIs in the contemporaneous network at T1 ........ 12
**Figure S5.** Bootstrapped difference tests of EIs in the contemporaneous network at T2 ........ 13
**Figure S6.** Bootstrapped difference tests of bridge EIs in the contemporaneous network at T1 ........ 14
**Figure S7.** Bootstrapped difference tests of bridge EIs in the contemporaneous network at T2 ........ 15
**Figure S8.** Stability of EI in the contemporaneous network at T1 ........ 16
**Figure S9.** Stability of EI in the contemporaneous network at T2 ........ 17
**Figure S10.** Stability of bridge EI in the contemporaneous network at T1 ........ 18
**Figure S11.** Stability of bridge EI in the contemporaneous network at T2 ........ 19
**Figure S12.** Bootstrapped 95% confidence intervals around each edge weight in the contemporaneous network at T1 ........ 20
**Figure S13.** Bootstrapped 95% confidence intervals around each edge weight in the contemporaneous network at T2 ........ 21
**Figure S14.** Bootstrapped difference tests of edges in the temporal network ........ 22
**Figure S15.** Bootstrapped difference tests of out-EIs in the temporal network ........ 23
**Figure S16.** Bootstrapped difference tests of in-EIs in the temporal network ........ 24
**Figure S17.** Stability of out-EI in the temporal network ........ 25
**Figure S18.** Stability of in-EI in the temporal network ........ 26
**Figure S19.** Bootstrapped 95% confidence intervals around each edge weight in the temporal network. ........ 27

**Table S1**

Demographic characteristics of the samples.

| | MCS full sample at wave 2 (N=15,778) | MCS full sample at wave 3 (N= 15,431) | Study Sample at wave 2 (N=3,750) | Study Sample at wave 3 (N=3,750) |
|---|---|---|---|---|
| CM age (M, SD) | 3.1 (0.2) | 5.2 (0.2) | 3.1 (0.2) | 5.2 (0.2) |
| OS age (M, SD) | 6.7 (2.1) | 8.5 (2.2) | 6.7 (2.2) | 8.2 (1.9) |
| Mother age (M, SD) | 31.9 (6.0) | 34 (6.0) | 34.7 (4.8) | 36.4 (4.7) |
| Father age (M, SD) | 35.2 (6.3) | 37.2 (6.4) | 37.2 (5.4) | 39.1 (5.4) |
| Sex constellation of siblings (%) | | | | |
| Sister pairs | / | / | 23.3 | / |
| Brother pairs | / | / | 26.3 | / |
| Older brother–younger sister pairs | / | / | 25.8 | / |
| Oder sister–younger brother pairs | / | / | 24.6 | / |
| Mother's education (%) | | | | |
| NVQ LEVEL 1 | 13.0 | 12.7 | 10.1 | 9.3 |
| NVQ LEVEL 2 | 41.0 | 41.2 | 38.8 | 29.7 |
| NVQ LEVEL 3 | 12.0 | 12.2 | 13.2 | 13.3 |
| NVQ LEVEL 4 | 29.0 | 29.1 | 32.4 | 32.3 |
| NVQ LEVEL 5 | 4.9 | 4.9 | 5.6 | 5.4 |
| Father's education (%) | | | | |
| NVQ LEVEL 1 | 7.6 | 11.9 | 10.8 | 11.5 |
| NVQ LEVEL 2 | 31.4 | 37.7 | 38.5 | 37.9 |
| NVQ LEVEL 3 | 17.4 | 9.8 | 10.5 | 9.9 |
| NVQ LEVEL 4 | 35.9 | 31.9 | 31.3 | 31.7 |
| NVQ LEVEL 5 | 7.6 | 8.6 | 9.0 | 9.1 |
| Social stratum | | | | |
| England—advantaged | 31.0 | / | 38.3 | / |
| England—disadvantaged | 23.3 | / | 18.5 | / |
| England—ethnic | 10.4 | / | 6.2 | / |
| Wales—advantaged | 5.5 | / | 7.0 | / |
| Wales—disadvantaged | 8.9 | / | 8.1 | / |
| Scotland—advantaged | 6.4 | / | 7.1 | / |
| Scotland—disadvantaged | 5.2 | / | 4.3 | / |
| Northern Ireland—advantaged | 4.0 | / | 5.4 | / |
| Northern Ireland—disadvantaged | 5.4 | / | 5.1 | / |
| Number of children in the family (M, SD) | 1.3 (0.5) | 1.3 (0.4) | 1.6 (0.9) | 1.7 (0.9) |

*Note*. CM = cohort member (younger sibling in this study); OS = older sibling; NVQ = National Vocational Qualification; *M* = mean; *SD* = standard deviation.

**Table S2**

Cronbach's Alphas for the Subscales of the Strengths and Difficulties Questionnaire.

| | Cronbach's alpha | |
|---|---|---|
| | Wave 2 | Wave 3 |
| Emotion symptom of older sibling | 0.67 | 0.71 |
| Conduct problem of older sibling | 0.61 | 0.61 |
| Hyperactivity of older sibling | 0.80 | 0.82 |
| Peer problem of older sibling | 0.59 | 0.61 |
| Pro-social of older sibling | 0.76 | 0.77 |
| Emotion symptom of younger sibling | 0.50 | 0.56 |
| Conduct problem of younger sibling | 0.66 | 0.50 |
| Hyperactivity of younger sibling | 0.71 | 0.76 |
| Peer problem of younger sibling | 0.59 | 0.61 |
| Pro-social of younger sibling | 0.66 | 0.69 |

**Table S3**

Descriptive statistics of variables.

| | *M* | | *(SD)* | | Min | | Max | | Skew | | Kurtosis | |
|---|---|---|---|---|---|---|---|---|---|---|---|---|
| | T1 | T2 | T1 | T2 | T1 | T2 | T1 | T2 | T1 | T2 | T1 | T2 |
| M1 | 1.43 | 1.39 | 0.73 | 0.73 | 1 | 1 | 5 | 5 | 1.80 | 2.13 | 3.15 | 4.64 |
| M2 | 1.34 | 1.30 | 0.66 | 0.66 | 1 | 1 | 5 | 5 | 2.16 | 2.59 | 5.01 | 7.40 |
| M3 | 1.56 | 1.55 | 0.79 | 0.81 | 1 | 1 | 5 | 5 | 1.48 | 1.55 | 2.23 | 2.34 |
| M4 | 1.80 | 1.73 | 0.86 | 0.88 | 1 | 1 | 5 | 5 | 1.02 | 1.21 | 0.88 | 1.32 |
| M5 | 1.29 | 1.29 | 0.66 | 0.68 | 1 | 1 | 5 | 5 | 2.58 | 2.74 | 7.13 | 7.97 |
| M6 | 1.47 | 1.50 | 0.73 | 0.76 | 1 | 1 | 5 | 5 | 1.60 | 1.66 | 2.48 | 2.89 |
| F1 | 1.39 | 1.38 | 0.72 | 0.74 | 1 | 1 | 5 | 5 | 1.97 | 2.22 | 3.97 | 5.23 |
| F2 | 1.26 | 1.28 | 0.60 | 0.64 | 1 | 1 | 5 | 5 | 2.62 | 2.64 | 7.80 | 7.51 |
| F3 | 1.77 | 1.78 | 0.85 | 0.90 | 1 | 1 | 5 | 5 | 0.92 | 1.07 | 0.42 | 0.82 |
| F4 | 1.79 | 1.77 | 0.86 | 0.88 | 1 | 1 | 5 | 5 | 0.99 | 1.11 | 0.73 | 0.97 |
| F5 | 1.22 | 1.24 | 0.57 | 0.63 | 1 | 1 | 5 | 5 | 3.00 | 3.09 | 9.88 | 10.3 |
| F6 | 1.45 | 1.52 | 0.70 | 0.75 | 1 | 1 | 5 | 5 | 1.62 | 1.47 | 2.52 | 2.13 |
| CM_ES | 6.21 | 6.16 | 1.32 | 1.40 | 5 | 5 | 14 | 15 | 1.48 | 1.67 | 2.86 | 3.68 |
| CM_PP | 6.32 | 9.40 | 1.44 | 0.98 | 5 | 5 | 14 | 15 | 1.17 | 0.98 | 1.18 | 3.12 |
| CM_CP | 7.58 | 7.45 | 1.92 | 1.03 | 5 | 5 | 15 | 13 | 0.72 | 0.98 | 0.24 | 2.03 |
| CM_H | 8.55 | 8.82 | 2.21 | 1.41 | 5 | 5 | 15 | 15 | 0.58 | 0.74 | -0.13 | 0.72 |
| CM_PS | 12.17 | 13.36 | 1.83 | 1.66 | 5 | 5 | 15 | 15 | -0.38 | -1.01 | -0.14 | 0.72 |
| OS_ES | 6.63 | 6.57 | 1.84 | 1.88 | 5 | 5 | 15 | 15 | 1.40 | 1.46 | 1.91 | 2.10 |
| OS_PP | 6.14 | 6.19 | 1.49 | 1.55 | 5 | 5 | 14 | 14 | 1.71 | 1.65 | 3.26 | 2.89 |
| OS_CP | 6.44 | 6.26 | 1.53 | 1.47 | 5 | 5 | 15 | 15 | 1.35 | 1.45 | 2.14 | 2.53 |
| OS_H | 7.93 | 7.55 | 2.55 | 2.47 | 5 | 5 | 15 | 15 | 0.83 | 1.06 | 0.01 | 0.62 |
| OS_PS | 13.56 | 13.71 | 1.74 | 1.72 | 5 | 5 | 15 | 15 | -1.38 | -1.51 | 1.94 | 2.13 |

*Note*. *M* = mean; *SD* = standard deviation; Min = minimum; Max = maximum; YS = younger sibling; OS = older sibling; ES = emotion symptom; CP = conduct problem; H = hyperactivity; PP = peer problem; PS = pro-social; M1 = mother felt depressed; M2 = mother felt hopeless; M3 = mother felt restless/fidgety; M4 = mother felt everything an effort; M5 = mother felt worthless; M6 = mother felt nervous; F1 = father felt depressed; F2 = father felt hopeless; F3 = father felt restless/fidgety; F4 = father felt everything an effort; F5 = father felt worthless; F6 = father felt nervous.

**Table S4**

Edge values in contemporaneous networks.

| | YS_ES | YS_PP | YS_CP | YS_H | YS_PS | OS_ES | OS_PP | OS_CP | OS_H | OS_PS | M1 | M2 | M3 | M4 | M5 | M6 | F1 | F2 | F3 | F4 | F5 | F6 |
|---|---|---|---|---|---|---|---|---|---|---|---|---|---|---|---|---|---|---|---|---|---|---|
| YS_ES | 1 | 0.219 | 0.086 | 0.063 | 0.037 | 0.103 | 0 | 0.045 | 0.039 | 0 | 0 | 0 | 0.014 | 0 | 0 | 0 | 0 | 0 | 0 | 0 | 0 | 0 |
| YS_PP | 0.234 | 1 | 0.017 | 0.059 | -0.205 | 0 | 0.156 | 0 | 0 | 0 | 0.028 | 0 | 0 | 0 | 0 | 0 | 0 | 0 | 0 | 0 | 0 | 0 |
| YS_CP | 0.103 | 0 | 1 | 0.31 | -0.193 | 0.043 | 0 | 0.091 | 0 | 0 | 0 | 0.046 | 0 | 0.015 | 0 | 0 | 0 | 0 | 0 | 0 | 0 | 0 |
| YS_H | 0.022 | 0.072 | 0.351 | 1 | -0.11 | 0.024 | 0 | 0 | 0 | 0 | 0.021 | 0 | 0.018 | 0 | 0 | 0 | 0 | 0 | 0 | 0 | 0 | 0 |
| YS_PS | 0 | -0.152 | -0.171 | -0.151 | 1 | 0 | 0 | 0.055 | 0 | 0.18 | 0 | 0 | 0 | 0 | 0 | 0 | 0 | 0 | 0 | 0 | 0 | 0 |
| OS_ES | 0.095 | 0 | 0 | 0.074 | 0 | 1 | 0.283 | 0.113 | 0.041 | 0.042 | 0.017 | 0 | 0.028 | 0.017 | 0 | 0.052 | 0 | 0 | 0 | 0 | 0 | 0 |
| OS_PP | 0 | 0.139 | 0 | 0 | 0 | 0.305 | 1 | 0.045 | 0.067 | -0.168 | 0.026 | 0 | 0 | 0 | 0 | 0 | 0 | 0 | 0 | 0 | 0 | 0 |
| OS_CP | 0.034 | 0 | 0.114 | 0 | 0.033 | 0.126 | 0.028 | 1 | 0.353 | -0.304 | 0 | 0 | 0 | 0 | 0 | 0 | 0 | 0 | 0 | 0 | 0 | 0 |
| OS_H | 0.03 | 0 | 0 | 0 | 0 | 0.062 | 0.086 | 0.354 | 1 | -0.16 | 0 | 0 | 0.033 | 0 | 0 | 0 | 0 | 0 | 0 | 0 | 0 | 0 |
| OS_PS | 0 | 0 | 0 | 0 | 0.162 | 0 | -0.138 | -0.282 | -0.133 | 1 | 0 | 0 | 0 | 0 | 0 | 0 | 0 | 0 | 0 | 0 | 0 | 0 |
| M1 | 0.034 | 0.027 | 0 | 0 | 0 | 0 | 0 | 0 | 0 | 0 | 1 | 0.346 | 0.099 | 0.14 | 0.189 | 0.039 | 0 | 0 | 0 | 0 | 0 | 0 |
| M2 | 0 | 0 | 0 | 0 | 0 | 0 | 0 | 0 | 0 | 0 | 0.359 | 1 | 0.069 | 0.109 | 0.358 | 0.092 | 0 | 0 | 0 | 0 | 0 | 0 |
| M3 | 0.029 | 0 | 0 | 0 | 0 | 0.023 | 0 | 0 | 0 | 0 | 0.154 | 0.067 | 1 | 0.235 | 0.068 | 0.144 | 0 | 0 | 0 | 0 | 0 | 0 |
| M4 | 0 | 0 | 0 | 0 | 0 | 0.036 | 0 | 0 | 0 | 0 | 0.118 | 0.096 | 0.203 | 1 | 0.132 | 0.062 | 0 | 0 | 0 | 0.057 | 0 | 0 |
| M5 | 0 | 0 | 0 | 0 | 0 | 0 | 0 | 0 | 0 | 0 | 0.148 | 0.398 | 0.032 | 0.132 | 1 | 0.146 | 0 | 0 | 0 | 0 | 0 | 0 |
| M6 | 0 | 0 | 0 | 0 | 0 | 0.046 | 0 | 0 | 0 | 0 | 0.102 | 0.057 | 0.185 | 0.09 | 0.15 | 1 | 0 | 0 | 0 | 0 | 0 | 0 |
| F1 | 0 | 0 | 0 | 0 | 0 | 0 | 0 | 0 | 0 | 0 | 0.043 | 0 | 0 | 0 | 0 | 0 | 1 | 0.327 | 0.072 | 0.173 | 0.144 | 0 |
| F2 | 0 | 0 | 0 | 0 | 0 | 0 | 0 | 0 | 0 | 0 | 0 | 0 | 0 | 0 | 0 | 0 | 0.357 | 1 | 0.04 | 0.07 | 0.381 | 0.121 |
| F3 | 0 | 0 | 0 | 0 | 0 | 0 | 0 | 0 | 0 | 0 | 0 | 0 | 0 | 0 | 0 | 0 | 0.106 | 0.063 | 1 | 0.289 | 0.068 | 0.171 |
| F4 | 0 | 0 | 0 | 0 | 0 | 0 | 0 | 0 | 0 | 0 | 0 | 0 | 0 | 0.051 | 0 | 0 | 0.12 | 0.12 | 0.276 | 1 | 0.076 | 0.094 |
| F5 | 0 | 0 | 0 | 0 | 0 | 0 | 0 | 0 | 0 | 0 | 0 | 0 | 0 | 0 | 0 | 0 | 0.132 | 0.408 | 0 | 0.122 | 1 | 0.093 |
| F6 | 0 | 0 | 0 | 0 | 0 | 0 | 0 | 0 | 0 | 0 | 0 | 0 | 0 | 0 | 0 | 0 | 0.106 | 0.035 | 0.188 | 0.089 | 0.108 | 1 |

*Note*. YS = younger sibling; OS = older sibling; ES = emotion symptom; CP = conduct problem; H = hyperactivity; PP = peer problem; PS = pro-social; M1 = mother felt depressed; M2 = mother felt hopeless; M3 = mother felt restless/fidgety; M4 = mother felt everything an effort; M5 = mother felt worthless; M6 = mother felt nervous; F1 = father felt depressed; F2 = father felt hopeless; F3 = father felt restless/fidgety; F4 = father felt everything an effort; F5 = father felt worthless; F6 = father felt nervous. Edge values above the diagonal represent partial correlations of the nodes at T1, while the values below the diagonal represent partial correlations of the nodes at T2. The intra-person edges were bolded, and edge values ≥ .09 were highlighted.

**Table S5**

Comparison of edge weights in contemporaneous networks at T1 and T2.

| | YS_ES | YS_PP | YS_CP | YS_H | YS_PS | OS_ES | OS_PP | OS_CP | OS_H | OS_PS | M1 | M2 | M3 | M4 | M5 | M6 | F1 | F2 | F3 | F4 | F5 |
|---|---|---|---|---|---|---|---|---|---|---|---|---|---|---|---|---|---|---|---|---|---|
| YS_PP | 0.006 | | | | | | | | | | | | | | | | | | | | |
| YS_CP | 0.068 | 0.012 | | | | | | | | | | | | | | | | | | | |
| YS_H | 0.034 | 0.004 | 0.166 | | | | | | | | | | | | | | | | | | |
| YS_PS | 0.058 | 0.246 | 0.174 | 0.101 | | | | | | | | | | | | | | | | | |
| OS_ES | 0.006 | 0.000 | 0.001 | 0.027 | 0.000 | | | | | | | | | | | | | | | | |
| OS_PP | 0.000 | 0.086 | 0.002 | 0.034 | 0.017 | 0.033 | | | | | | | | | | | | | | | |
| OS_CP | 0.008 | 0.018 | 0.008 | 0.000 | 0.000 | 0.014 | 0.012 | | | | | | | | | | | | | | |
| OS_H | 0.002 | 0.011 | 0.001 | 0.006 | 0.000 | 0.022 | 0.023 | 0.004 | | | | | | | | | | | | | |
| OS_PS | 0.000 | 0.040 | 0.019 | 0.010 | 0.063 | 0.000 | 0.007 | 0.011 | 0.009 | | | | | | | | | | | | |
| M1 | 0.022 | 0.021 | 0.005 | 0.013 | 0.000 | 0.019 | 0.024 | 0.000 | 0.004 | 0.000 | | | | | | | | | | | |
| M2 | 0.020 | 0.000 | 0.053 | 0.000 | 0.021 | 0.000 | 0.012 | 0.015 | 0.021 | 0.000 | 0.016 | | | | | | | | | | |
| M3 | 0.021 | 0.004 | 0.008 | 0.016 | 0.000 | 0.008 | 0.010 | 0.016 | 0.001 | 0.000 | 0.052 | 0.002 | | | | | | | | | |
| M4 | 0.010 | 0.003 | 0.029 | 0.000 | 0.031 | 0.017 | 0.010 | 0.023 | 0.009 | 0.000 | 0.018 | 0.014 | 0.023 | | | | | | | | |
| M5 | 0.000 | 0.000 | 0.000 | 0.000 | 0.000 | 0.004 | 0.021 | 0.006 | 0.000 | 0.000 | 0.034 | 0.044 | 0.029 | 0.004 | | | | | | | |
| M6 | 0.005 | 0.000 | 0.007 | 0.000 | 0.000 | 0.004 | 0.000 | 0.000 | 0.000 | 0.000 | 0.057 | 0.031 | 0.043 | 0.029 | 0.010 | | | | | | |
| F1 | 0.005 | 0.000 | 0.002 | 0.022 | 0.000 | 0.000 | 0.010 | 0.009 | 0.009 | 0.000 | 0.040 | 0.000 | 0.008 | 0.000 | 0.000 | 0.000 | | | | | |
| F2 | 0.000 | 0.009 | 0.005 | 0.013 | 0.000 | 0.011 | 0.019 | 0.000 | 0.000 | 0.000 | 0.005 | 0.000 | 0.000 | 0.000 | 0.000 | 0.000 | 0.026 | | | | |
| F3 | 0.000 | 0.000 | 0.002 | 0.000 | 0.000 | 0.000 | 0.000 | 0.000 | 0.000 | 0.000 | 0.000 | 0.000 | 0.000 | 0.000 | 0.000 | 0.000 | 0.030 | 0.018 | | | |
| F4 | 0.016 | 0.020 | 0.002 | 0.008 | 0.000 | 0.000 | 0.007 | 0.000 | 0.017 | 0.000 | 0.000 | 0.000 | 0.000 | 0.004 | 0.000 | 0.000 | 0.050 | 0.044 | 0.003 | | |
| F5 | 0.000 | 0.015 | 0.000 | 0.000 | 0.000 | 0.000 | 0.004 | 0.009 | 0.000 | 0.000 | 0.002 | 0.003 | 0.000 | 0.000 | 0.010 | 0.000 | 0.010 | 0.029 | 0.054 | 0.050 | |
| F6 | 0.000 | 0.000 | 0.000 | 0.000 | 0.000 | 0.000 | 0.000 | 0.000 | 0.000 | 0.000 | 0.000 | 0.000 | 0.000 | 0.000 | 0.000 | 0.008 | 0.068 | 0.080 | 0.020 | 0.001 | 0.017 |

*Note*. YS = younger sibling; OS = older sibling; ES = emotion symptom; CP = conduct problem; H = hyperactivity; PP = peer problem; PS = pro-social; M1 = mother felt depressed; M2 = mother felt hopeless; M3 = mother felt restless/fidgety; M4 = mother felt everything an effort; M5 = mother felt worthless; M6 = mother felt nervous; F1 = father felt depressed; F2 = father felt hopeless; F3 = father felt restless/fidgety; F4 = father felt everything an effort; F5 = father felt worthless; F6 = father felt nervous. Values represent edge differences in the contemporaneous networks at T1 and T2. Highlighted values indicate $p \leq 0.05$ for edge-wise comparisons.

**Table S6**

Edge values in temporal networks.

| | YS_ES_T2 | YS_PP_T2 | YS_CP_T2 | YS_H_T2 | YS_PS_T2 | OS_ES_T2 | OS_PP_T2 | OS_CP_T2 | OS_H_T2 | OS_PS_T2 |
|---|---|---|---|---|---|---|---|---|---|---|
| YS_ES_T1 | **0.363** | **0.073** | **0.033** | **0.048** | **0** | 0.017 | 0 | 0.035 | 0 | 0 |
| YS_PP_T1 | **0.071** | **0.093** | **0.017** | **0.037** | **-0.067** | 0 | 0.036 | 0.018 | 0 | -0.013 |
| YS_CP_T1 | **0.017** | **-0.01** | **0.13** | **0.043** | **-0.049** | 0.026 | 0.022 | 0.042 | 0.004 | -0.017 |
| YS_H_T1 | **0.021** | **0.014** | **0.002** | **0.158** | **-0.044** | 0.011 | 0.002 | -0.019 | -0.032 | 0.02 |
| YS_PS_T1 | **0** | **0.051** | **0.032** | **0.088** | **0.324** | 0 | 0 | 0 | 0.01 | 0.031 |
| OS_ES_T1 | 0.048 | 0.004 | 0.005 | 0.051 | -0.004 | **0.459** | **0.014** | **-0.016** | **0** | **0** |
| OS_PP_T1 | 0 | 0.043 | 0.018 | 0.031 | 0.041 | **0.093** | **0.488** | **0.017** | **0.043** | **-0.048** |
| OS_CP_T1 | 0.017 | 0.017 | 0.024 | 0 | 0.005 | **0.021** | **0.037** | **0.44** | **0.109** | **-0.089** |
| OS_H_T1 | 0.023 | 0.003 | 0 | 0.008 | 0 | **0.023** | **0.032** | **0.055** | **0.589** | **-0.019** |
| OS_PS_T1 | 0 | 0.035 | 0 | 0.037 | 0.097 | **0.02** | **-0.019** | **-0.038** | **-0.038** | **0.461** |
| M1_T1 | 0.004 | 0.012 | 0.022 | 0.001 | 0 | 0.076 | 0.106 | 0.063 | 0.126 | -0.095 |
| M2_T1 | 0.097 | 0 | 0 | 0.063 | 0 | 0.005 | 0 | -0.007 | 0 | 0.063 |
| M3_T1 | 0.009 | 0.058 | 0.002 | 0.01 | 0 | 0.072 | 0 | 0.026 | 0 | 0.017 |
| M4_T1 | 0.006 | 0 | 0.031 | 0.059 | 0 | 0.036 | 0 | 0 | 0.012 | 0 |
| M5_T1 | 0.089 | 0.016 | 0.036 | 0 | -0.041 | 0.03 | 0.009 | -0.016 | 0 | 0 |
| M6_T1 | 0 | 0.013 | 0 | -0.039 | -0.02 | 0.026 | 0 | 0.026 | 0 | 0 |
| F1_T1 | 0.018 | 0.025 | 0 | 0.06 | 0 | 0 | 0 | 0 | 0 | 0 |
| F2_T1 | 0 | -0.013 | 0 | 0 | 0 | 0 | 0.093 | 0.007 | 0 | 0 |
| F3_T1 | 0 | 0 | 0 | 0.034 | 0 | 0.022 | 0 | 0.027 | 0 | 0 |
| F4_T1 | 0.011 | 0.023 | 0 | 0 | -0.006 | 0.003 | 0.009 | -0.036 | 0 | 0.001 |
| F5_T1 | 0 | 0 | 0 | 0.007 | 0.031 | 0.021 | 0.018 | 0.048 | 0 | 0 |
| F6_T1 | 0 | 0.005 | 0 | 0 | 0 | -0.038 | 0 | -0.021 | -0.017 | 0.011 |

**Table S6 (Continued)**

Edge values in temporal networks.

| | M1_T2 | M2_T2 | M3_T2 | M4_T2 | M5_T2 | M6_T2 | F1_T2 | F2_T2 | F3_T2 | F4_T2 | F5_T2 | F6_T2 |
|---|---|---|---|---|---|---|---|---|---|---|---|---|
| YS_ES_T1 | 0.015 | 0.005 | 0.004 | 0.012 | 0 | 0.008 | 0.013 | 0.013 | -0.001 | 0 | 0.003 | 0 |
| YS_PP_T1 | 0.027 | 0.009 | 0.024 | 0.022 | 0.015 | 0.008 | 0.007 | 0.01 | -0.006 | 0.018 | 0 | 0 |
| YS_CP_T1 | 0.004 | 0.002 | 0 | 0 | 0 | 0 | 0.015 | 0.011 | 0.011 | 0.002 | 0.004 | 0 |
| YS_H_T1 | 0.004 | 0 | 0.017 | 0.002 | 0 | 0 | 0 | 0.001 | -0.001 | 0.004 | 0.005 | 0 |
| YS_PS_T1 | 0.015 | 0 | 0.001 | 0 | 0.008 | 0 | 0.001 | 0.001 | 0 | 0 | 0.001 | 0 |
| OS_ES_T1 | 0.016 | 0.003 | 0.031 | 0.008 | 0.007 | 0.012 | 0.008 | 0.004 | 0.011 | 0.012 | 0 | 0.001 |
| OS_PP_T1 | 0.005 | 0.006 | 0.004 | 0 | 0 | 0 | 0 | 0.008 | 0 | 0 | 0 | 0 |
| OS_CP_T1 | 0.023 | 0.018 | 0.022 | 0.023 | 0.016 | 0.004 | 0 | 0 | 0 | 0.001 | 0 | 0 |
| OS_H_T1 | -0.005 | 0 | 0.005 | 0 | 0 | 0 | 0 | 0 | 0.006 | 0.007 | 0.006 | 0 |
| OS_PS_T1 | -0.002 | 0 | 0 | -0.003 | -0.008 | 0 | 0 | 0 | 0 | -0.001 | 0 | -0.002 |
| M1_T1 | **0.17** | **0.072** | **0.038** | **0.065** | **0.068** | **0.001** | 0.03 | 0.02 | 0.033 | 0.03 | 0.023 | 0 |
| M2_T1 | **0.105** | **0.155** | **0.078** | **0.035** | **0.072** | **0.052** | 0.051 | 0.029 | 0.029 | 0 | 0.021 | 0.002 |
| M3_T1 | **0.063** | **0.055** | **0.233** | **0.059** | **0.041** | **0.066** | 0.016 | 0 | 0.04 | 0 | 0 | 0.007 |
| M4_T1 | **0.042** | **0.016** | **0.037** | **0.282** | **0.03** | **0.061** | 0 | 0.015 | 0.01 | 0.088 | 0.028 | 0 |
| M5_T1 | **0.065** | **0.114** | **0** | **0.009** | **0.226** | **0.048** | 0.034 | 0.036 | 0.017 | 0.02 | 0.051 | 0 |
| M6_T1 | **0.009** | **0.024** | **0.055** | **0.022** | **0.026** | **0.25** | 0 | 0 | 0.03 | 0 | 0 | 0.085 |
| F1_T1 | 0.056 | 0.012 | 0.026 | 0 | 0 | 0.001 | **0.219** | **0.076** | **0** | **0.058** | **0.047** | **0.035** |
| F2_T1 | 0.007 | 0.025 | 0 | 0.011 | 0.032 | 0 | **0.049** | **0.134** | **0.067** | **0** | **0.066** | **0** |
| F3_T1 | 0.014 | 0 | 0.018 | 0.013 | 0 | 0.011 | **0.012** | **0.002** | **0.328** | **0.06** | **0** | **0.036** |
| F4_T1 | -0.002 | 0 | 0 | 0.088 | -0.015 | 0 | **0.051** | **0.033** | **0.049** | **0.258** | **0.031** | **0.038** |
| F5_T1 | 0.051 | 0.017 | 0.051 | 0.013 | 0.048 | 0 | **0.085** | **0.17** | **0** | **0.05** | **0.256** | **0.059** |
| F6_T1 | 0.02 | 0.027 | 0.022 | 0.018 | 0.052 | 0.103 | **0.048** | **0.024** | **0.076** | **0.048** | **0.045** | **0.297** |

*Note*. YS = younger sibling; OS = older sibling; ES = emotion symptom; CP = conduct problem; H = hyperactivity; PP = peer problem; PS = pro-social; M1 = mother felt depressed; M2 = mother felt hopeless; M3 = mother felt restless/fidgety; M4 = mother felt everything an effort; M5 = mother felt worthless; M6 = mother felt nervous; F1 = father felt depressed; F2 = father felt hopeless; F3 = father felt restless/fidgety; F4 = father felt everything an effort; F5 = father felt worthless; F6 = father felt nervous; T1= time 1; T2= time 2. The intra-person edges were bolded, and edge values ≥ .09 were highlighted.

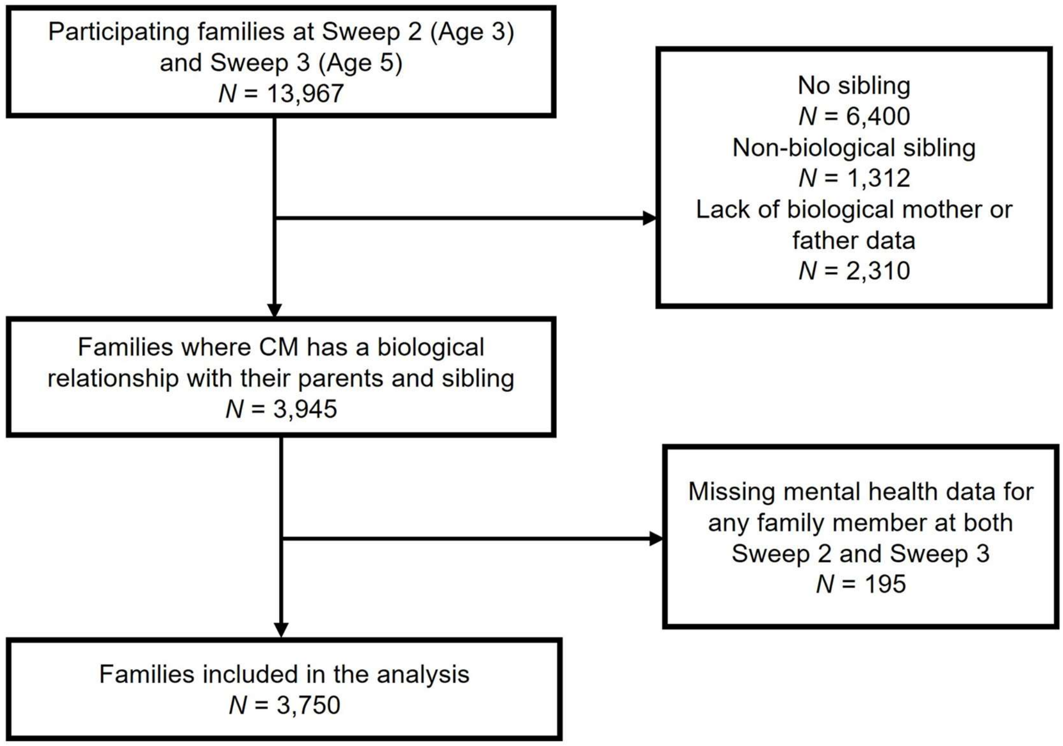


**Figure S1.** Flow chart of participants.

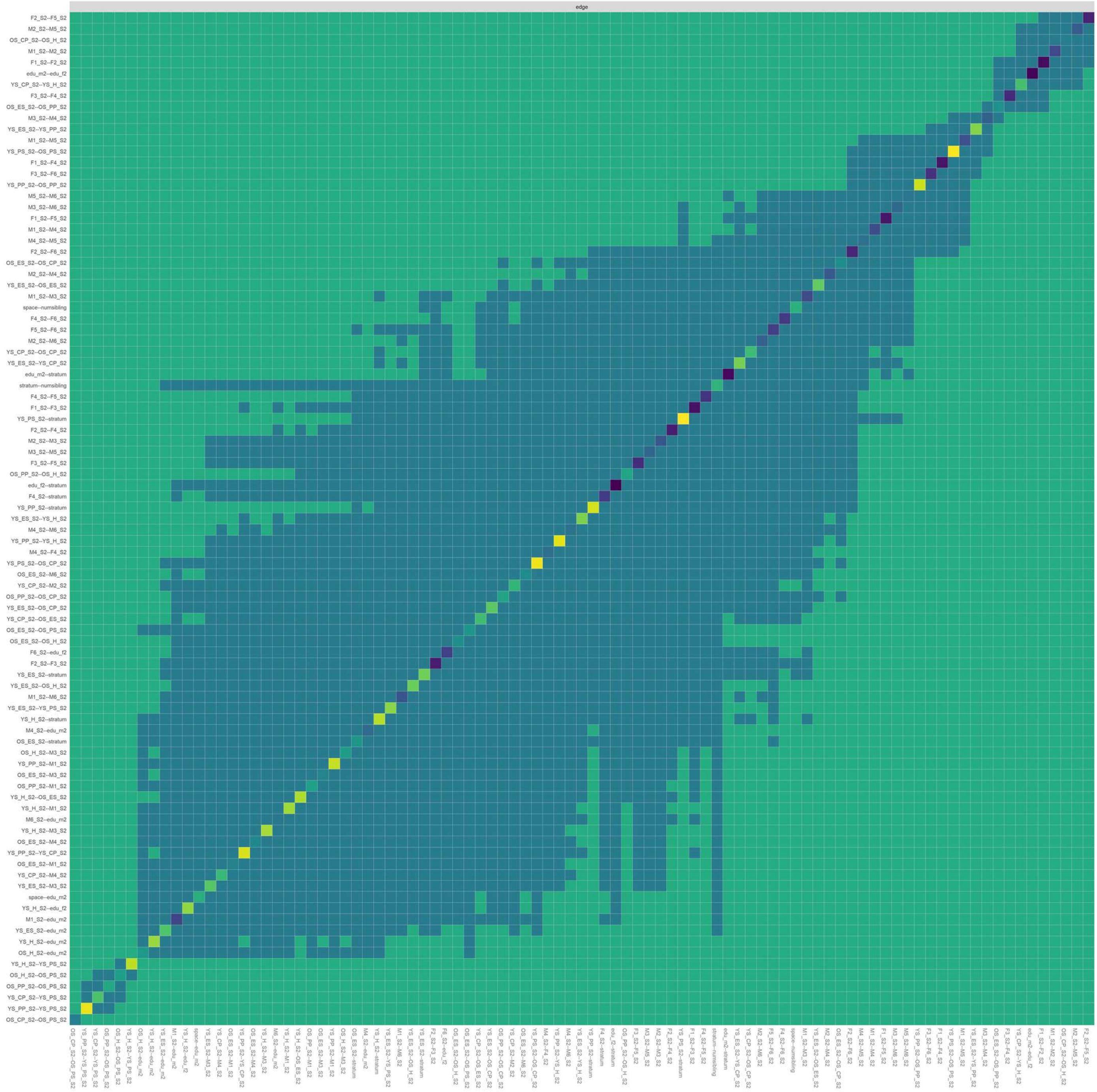


**Figure S2.** Bootstrapped difference tests of edges in the contemporaneous network at T1. YS = younger sibling; OS = older sibling; ES = emotion symptom; CP = conduct problem; H = hyperactivity; PP = peer problem; PS = pro-social; M1 = mother felt depressed; M2 = mother felt hopeless; M3 = mother felt restless/fidgety; M4 = mother felt everything an effort; M5 = mother felt worthless; M6 = mother felt nervous; F1 = father felt depressed; F2 = father felt hopeless; F3 = father felt restless/fidgety; F4 = father felt everything an effort; F5 = father felt worthless; F6 = father felt nervous; stratum = social stratum; space = age spacing between siblings; sexcon = sex constellation of siblings; edu_m = educational level of mother; edu_f = educational level of father; numchild = number of child within the family. Blue boxes indicate edges that do not differ significantly from one another and green boxes represent edges that do differ significantly from one another. The color of the boxes on the diagonal corresponds to the edge values, with darker colors representing higher edge values and lighter colors representing lower edge values.

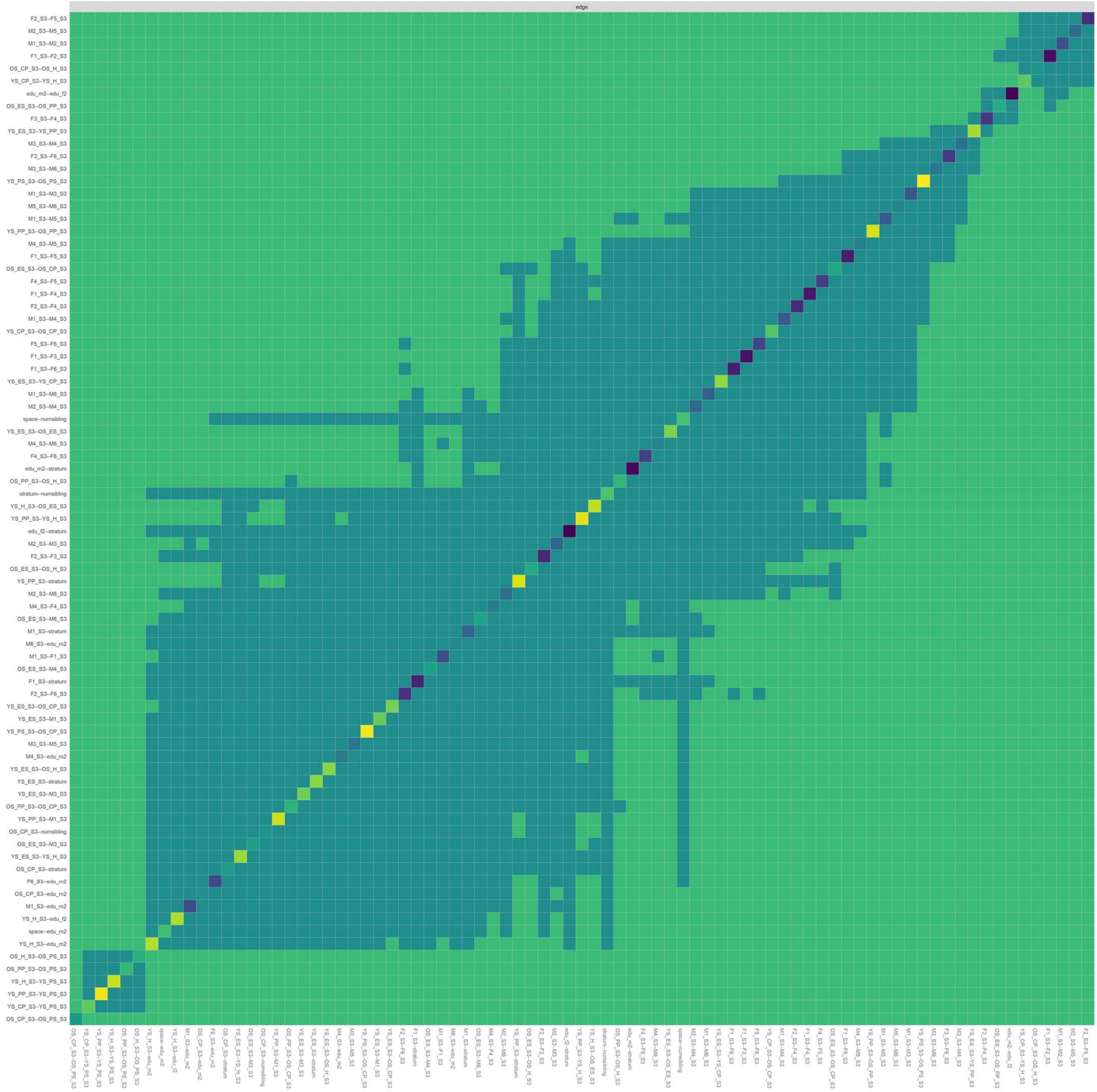


**Figure S3.** Bootstrapped difference tests of edges in the contemporaneous network at T2. YS = younger sibling; OS = older sibling; ES = emotion symptom; CP = conduct problem; H = hyperactivity; PP = peer problem; PS = pro-social; M1 = mother felt depressed; M2 = mother felt hopeless; M3 = mother felt restless/fidgety; M4 = mother felt everything an effort; M5 = mother felt worthless; M6 = mother felt nervous; F1 = father felt depressed; F2 = father felt hopeless; F3 = father felt restless/fidgety; F4 = father felt everything an effort; F5 = father felt worthless; F6 = father felt nervous; stratum = social stratum; space = age spacing between siblings; sexcon = sex constellation of siblings; edu_m = educational level of mother; edu_f = educational level of father; numchild = number of child within the family. Blue boxes indicate edges that do not differ significantly from one another and green boxes represent edges that do differ significantly from one another. The color of the boxes on the diagonal corresponds to the edge values, with darker colors representing higher edge values and lighter colors representing lower edge values.

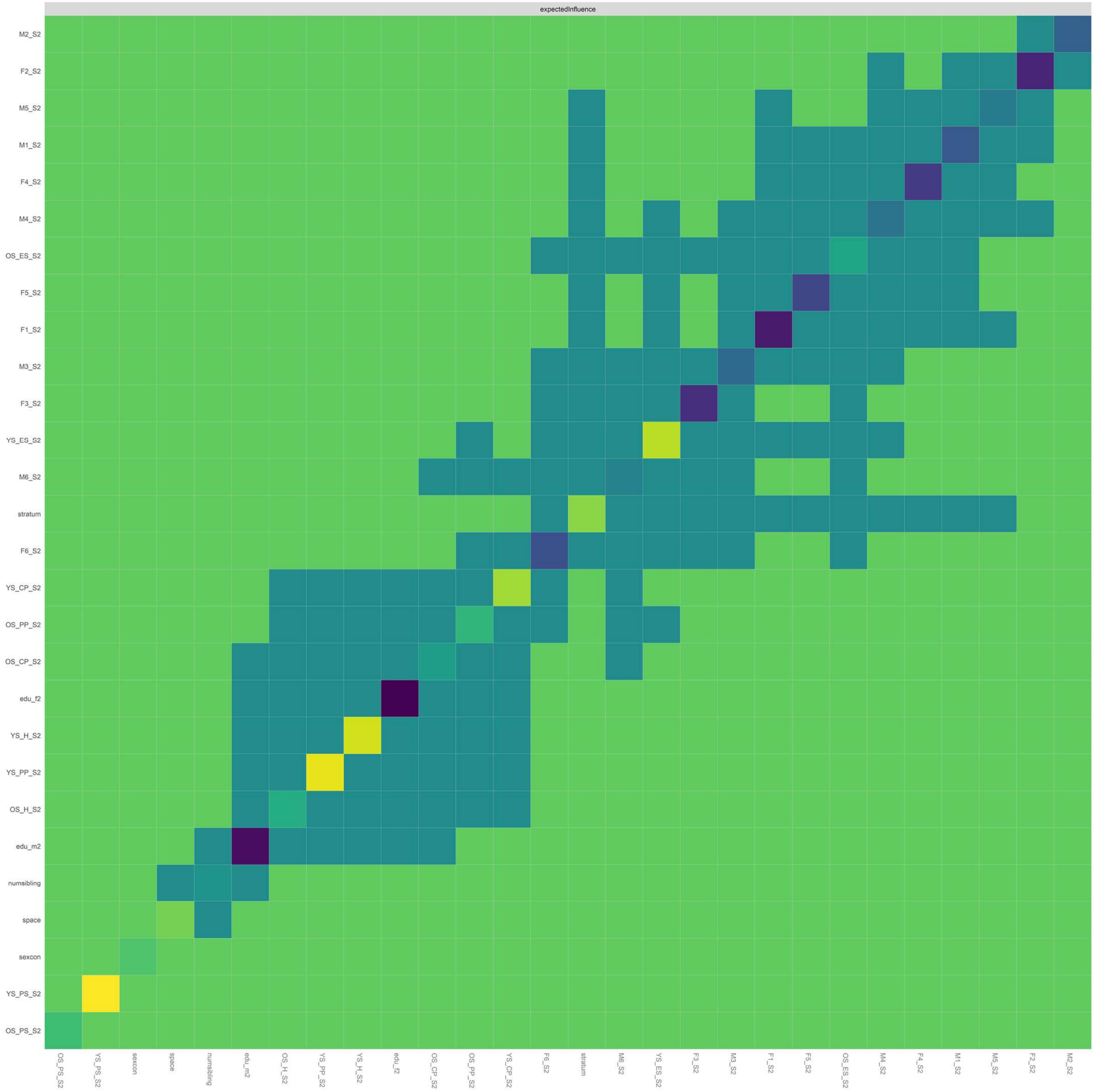


**Figure S4.** Bootstrapped difference tests of EIs in the contemporaneous network at T1. YS = younger sibling; OS = older sibling; ES = emotion symptom; CP = conduct problem; H = hyperactivity; PP = peer problem; PS = pro-social; M1 = mother felt depressed; M2 = mother felt hopeless; M3 = mother felt restless/fidgety; M4 = mother felt everything an effort; M5 = mother felt worthless; M6 = mother felt nervous; F1 = father felt depressed; F2 = father felt hopeless; F3 = father felt restless/fidgety; F4 = father felt everything an effort; F5 = father felt worthless; F6 = father felt nervous; stratum = social stratum; space = age spacing between siblings; sexcon = sex constellation of siblings; edu_m = educational level of mother; edu_f = educational level of father; numchild = number of child within the family. Blue boxes indicate nodes that do not differ significantly from one another and green boxes represent nodes that do differ significantly from one another. The color of the boxes on the diagonal corresponds to the value of node EI, with darker colors representing higher EI and lighter colors representing lower EI.

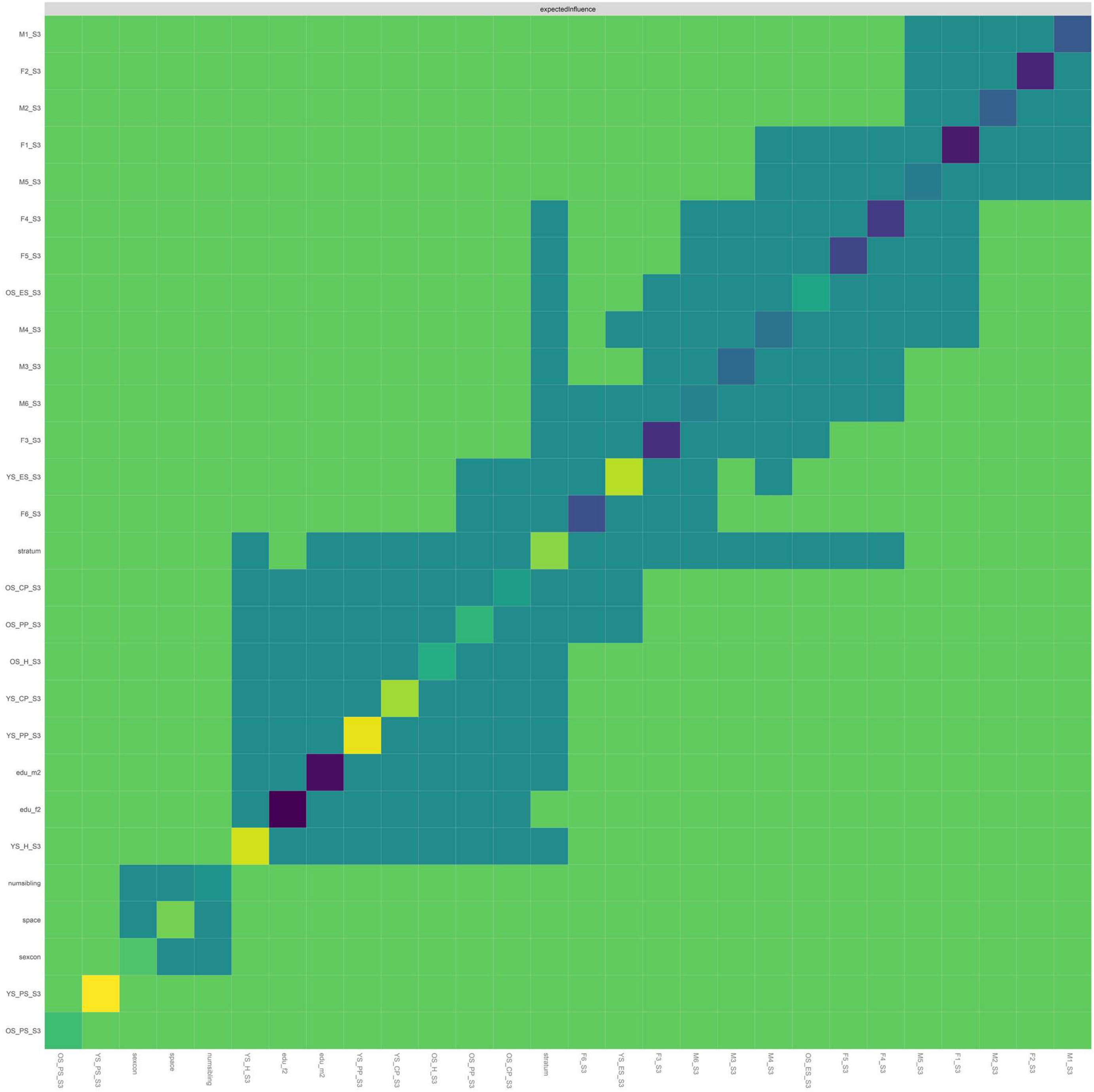


**Figure S5.** Bootstrapped difference tests of EIs in the contemporaneous network at T2. YS = younger sibling; OS = older sibling; ES = emotion symptom; CP = conduct problem; H = hyperactivity; PP = peer problem; PS = pro-social; M1 = mother felt depressed; M2 = mother felt hopeless; M3 = mother felt restless/fidgety; M4 = mother felt everything an effort; M5 = mother felt worthless; M6 = mother felt nervous; F1 = father felt depressed; F2 = father felt hopeless; F3 = father felt restless/fidgety; F4 = father felt everything an effort; F5 = father felt worthless; F6 = father felt nervous; stratum = social stratum; space = age spacing between siblings; sexcon = sex constellation of siblings; edu_m = educational level of mother; edu_f = educational level of father; numchild = number of child within the family. Blue boxes indicate nodes that do not differ significantly from one another and green boxes represent nodes that do differ significantly from one another. The color of the boxes on the diagonal corresponds to the value of node EI, with darker colors representing higher EI and lighter colors representing lower EI.

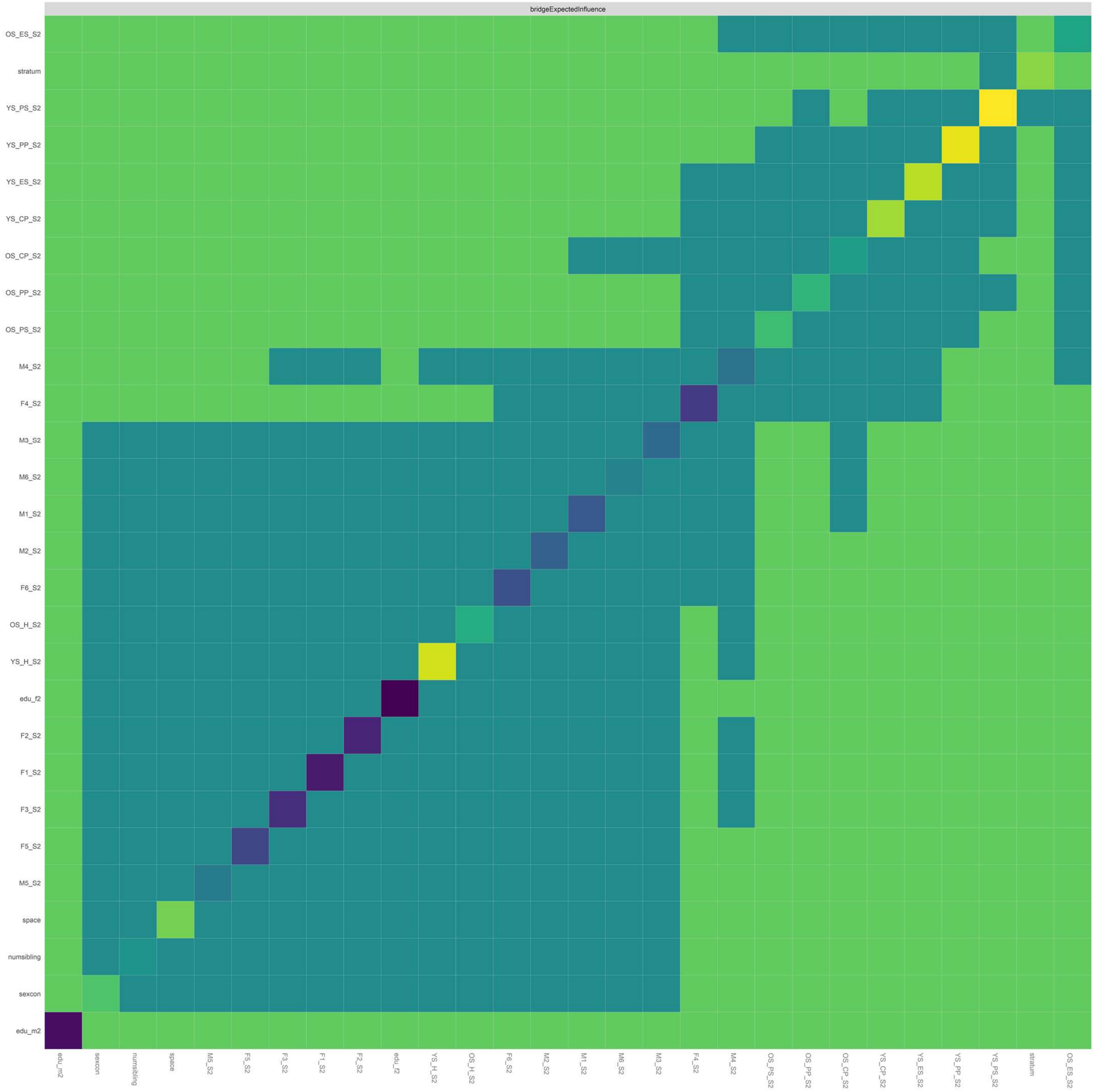


**Figure S6.** Bootstrapped difference tests of bridge EIs in the contemporaneous network at T1. YS = younger sibling; OS = older sibling; ES = emotion symptom; CP = conduct problem; H = hyperactivity; PP = peer problem; PS = pro-social; M1 = mother felt depressed; M2 = mother felt hopeless; M3 = mother felt restless/fidgety; M4 = mother felt everything an effort; M5 = mother felt worthless; M6 = mother felt nervous; F1 = father felt depressed; F2 = father felt hopeless; F3 = father felt restless/fidgety; F4 = father felt everything an effort; F5 = father felt worthless; F6 = father felt nervous; stratum = social stratum; space = age spacing between siblings; sexcon = sex constellation of siblings; edu_m = educational level of mother; edu_f = educational level of father; numchild = number of child within the family. Blue boxes indicate nodes that do not differ significantly from one another and green boxes represent nodes that do differ significantly from one another. The color of the boxes on the diagonal corresponds to the value of node bridge EIs, with darker colors representing higher bridge EIs and lighter colors representing lower bridge EIs.

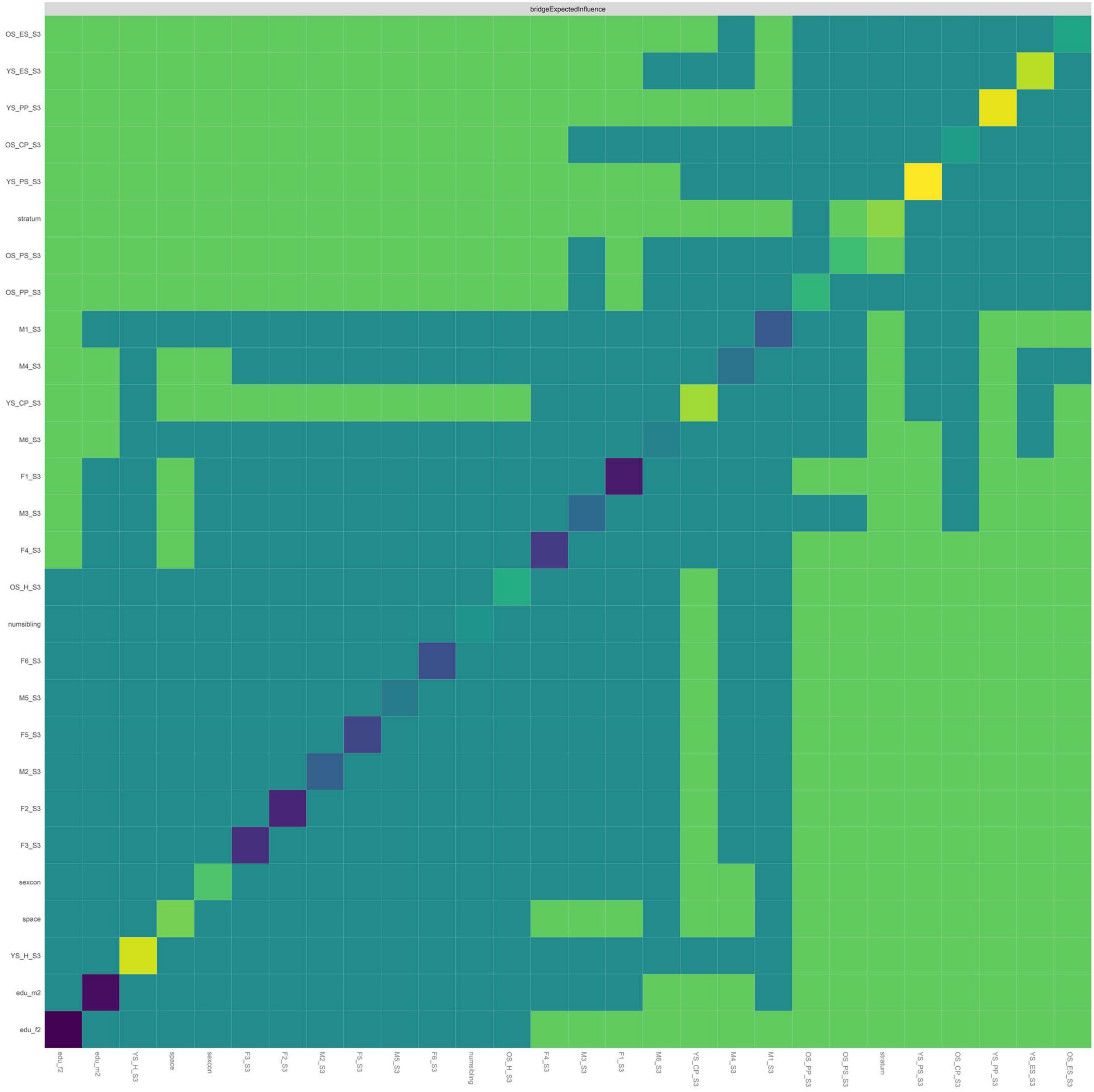


**Figure S7.** Bootstrapped difference tests of bridge EIs in the contemporaneous network at T2. YS = younger sibling; OS = older sibling; ES = emotion symptom; CP = conduct problem; H = hyperactivity; PP = peer problem; PS = pro-social; M1 = mother felt depressed; M2 = mother felt hopeless; M3 = mother felt restless/fidgety; M4 = mother felt everything an effort; M5 = mother felt worthless; M6 = mother felt nervous; F1 = father felt depressed; F2 = father felt hopeless; F3 = father felt restless/fidgety; F4 = father felt everything an effort; F5 = father felt worthless; F6 = father felt nervous; stratum = social stratum; space = age spacing between siblings; sexcon = sex constellation of siblings; edu_m = educational level of mother; edu_f = educational level of father; numchild = number of child within the family. Blue boxes indicate nodes that do not differ significantly from one another and green boxes represent nodes that do differ significantly from one another. The color of the boxes on the diagonal corresponds to the value of node bridge EIs, with darker colors representing higher bridge EIs and lighter colors representing lower bridge EIs.

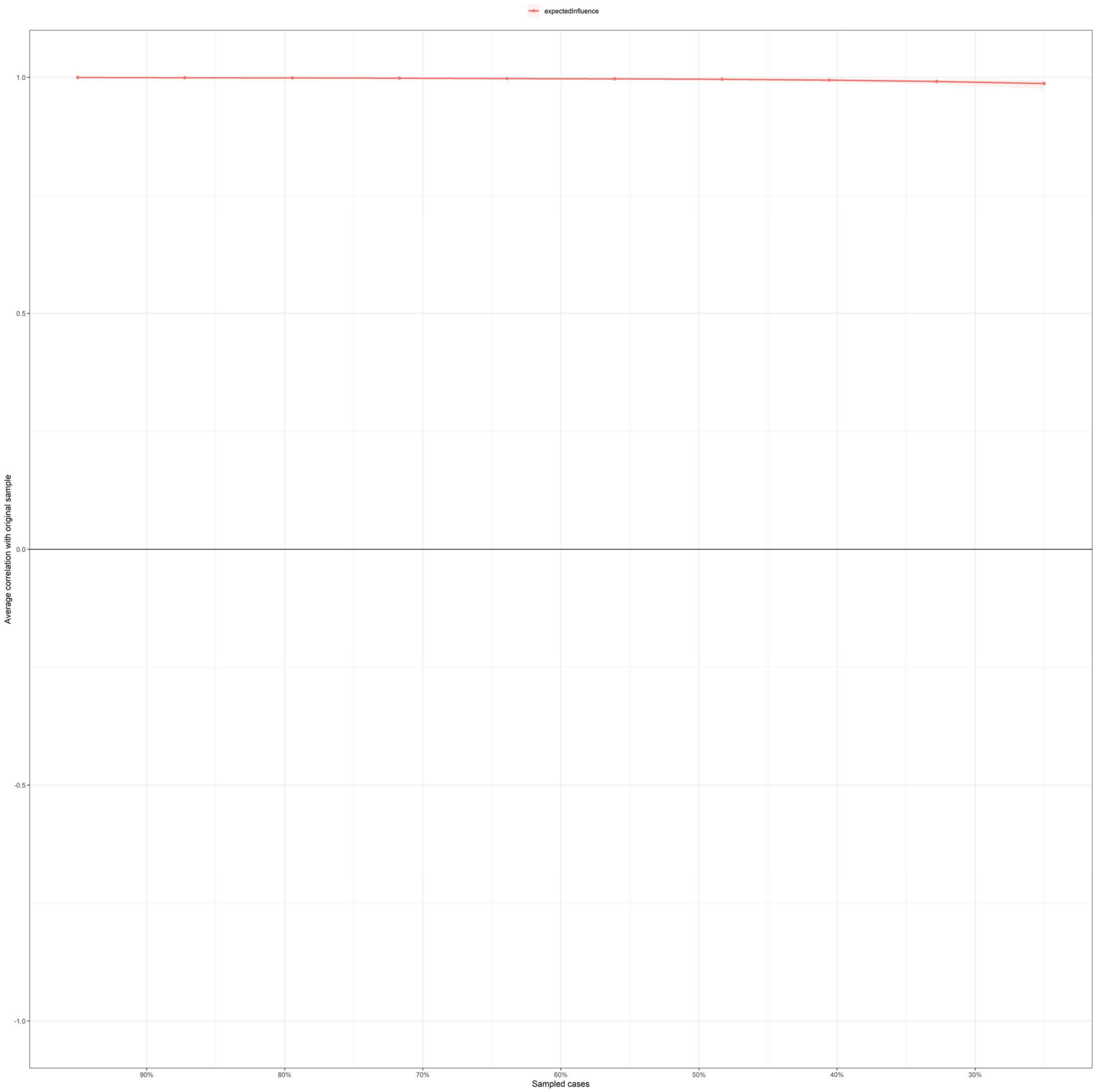


**Figure S8.** Stability of EI in the contemporaneous network at T1.

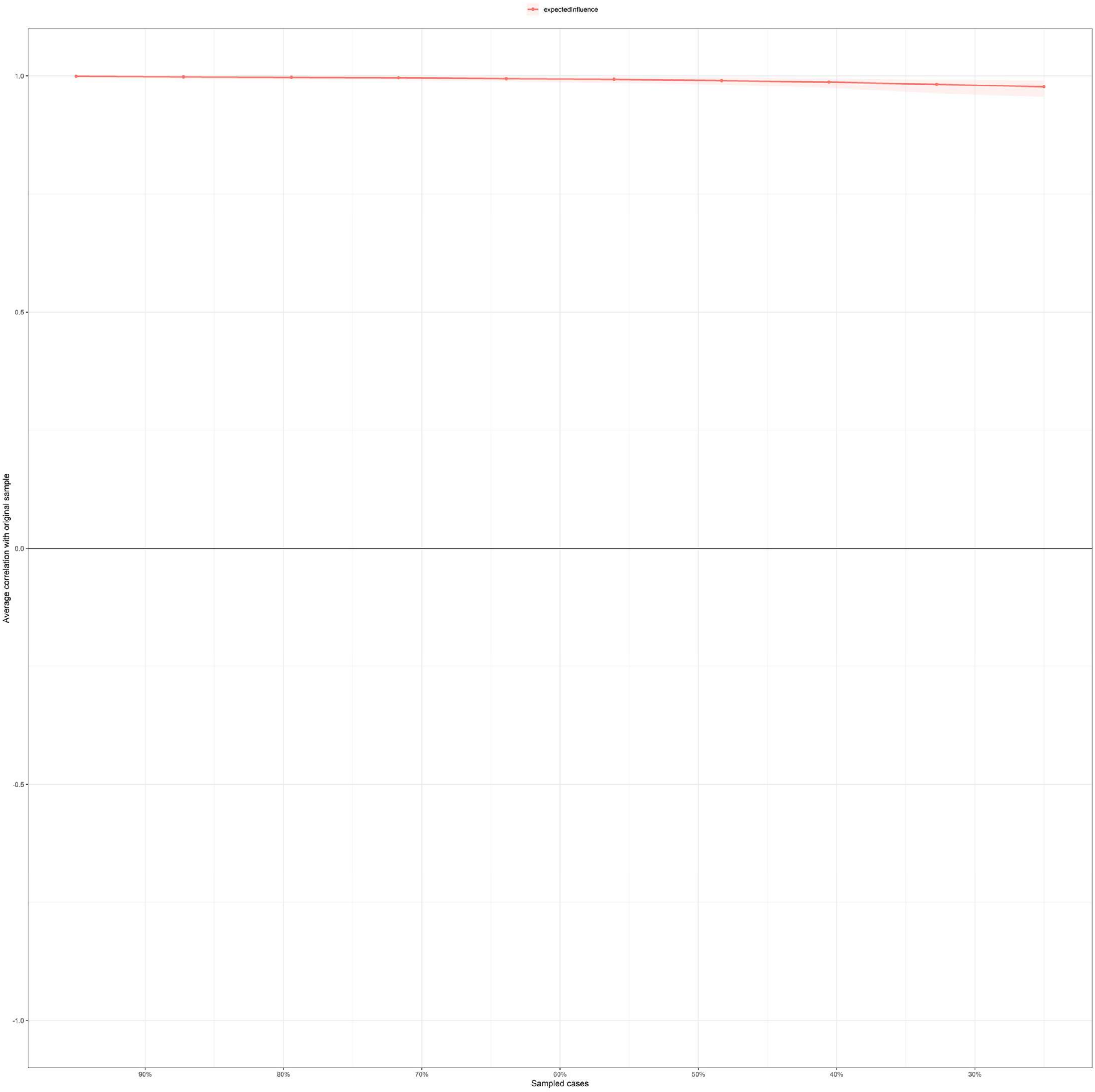


**Figure S9.** Stability of EI in the contemporaneous network at T2.

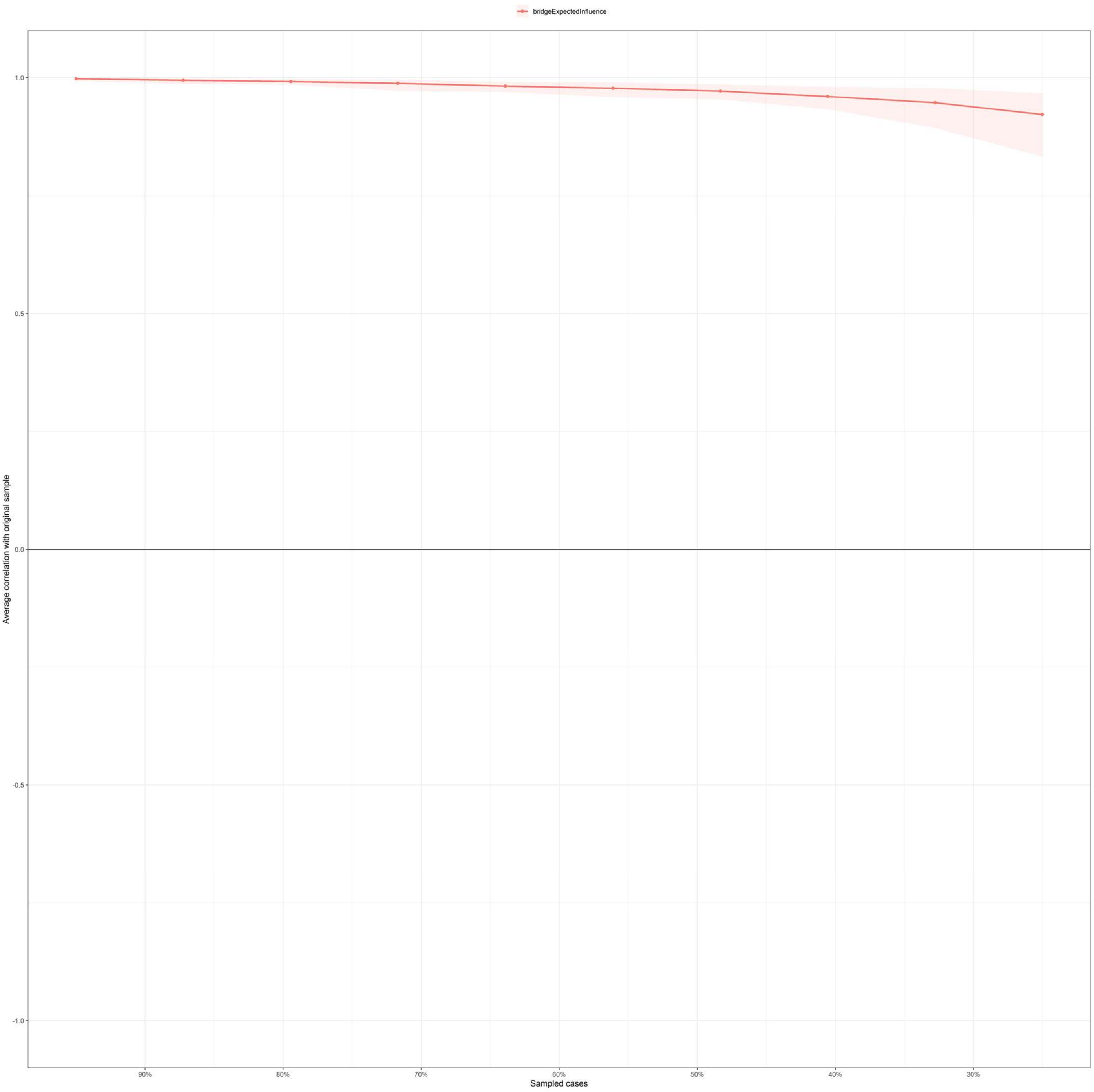


**Figure S10.** Stability of bridge EI in the contemporaneous network at T1.

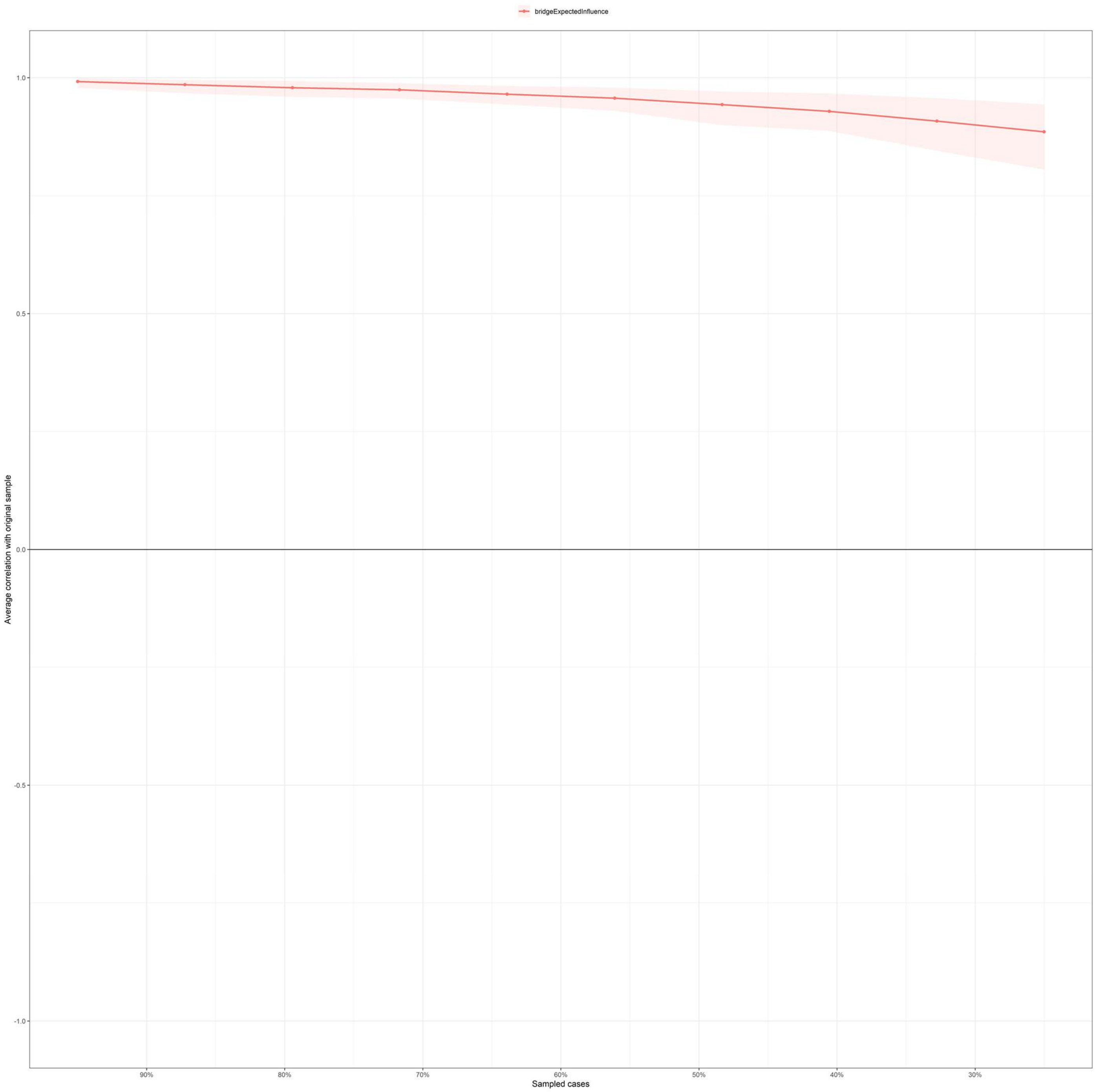


**Figure S11.** Stability of bridge EI in the contemporaneous network at T2.

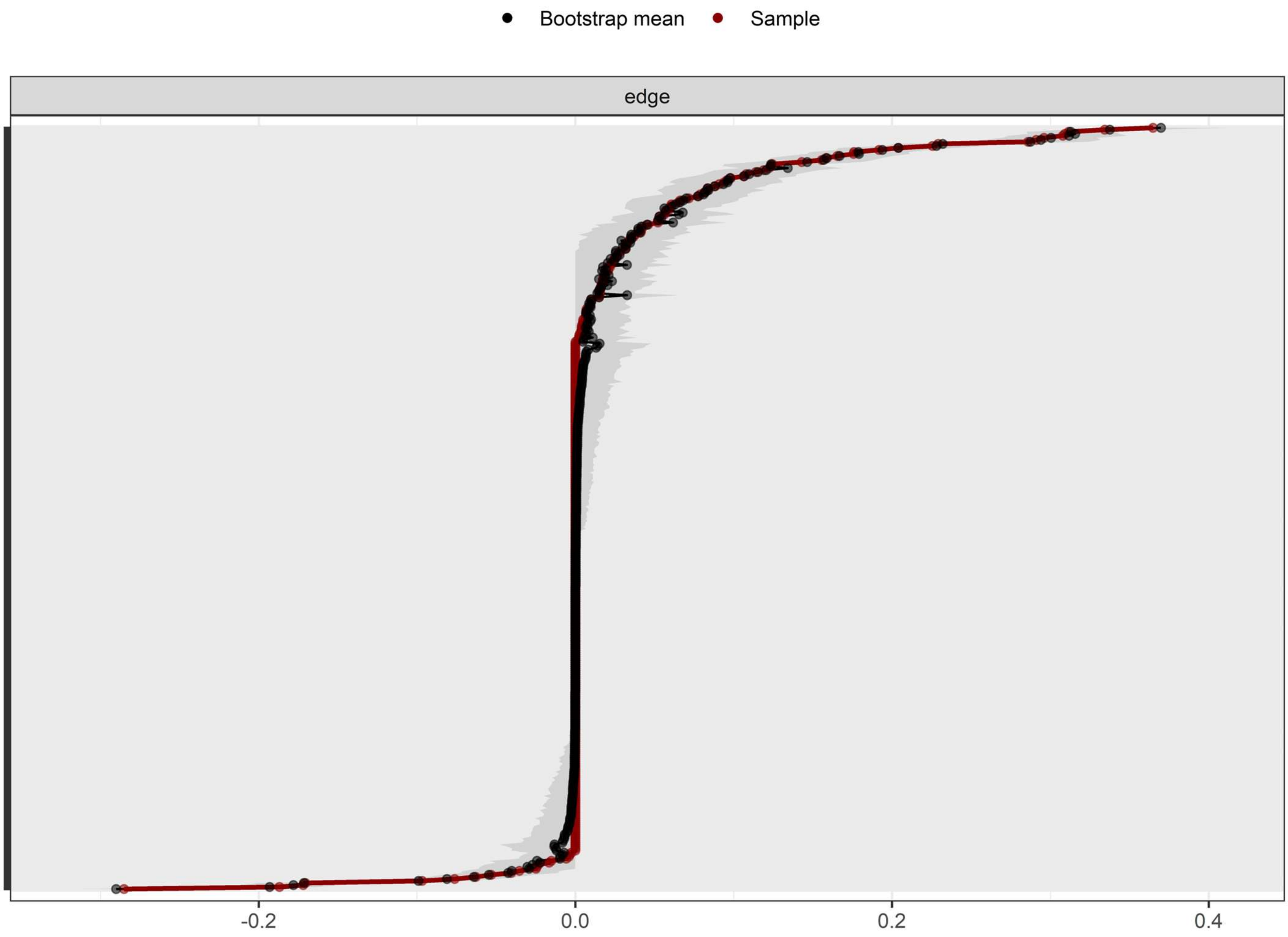


**Figure S12.** Bootstrapped 95% confidence intervals around each edge weight in the contemporaneous network at T1. YS = younger sibling; OS = older sibling; ES = emotion Symptoms; CP = conduct problem; H = hyperactivity; PP = peer problem; PS = pro-social; M1 = mother felt depressed; M2 = mother felt hopeless; M3 = mother felt restless/fidgety; M4 = mother felt everything an effort; M5 = mother felt worthless; M6 = mother felt nervous; F1 = father felt depressed; F2 = father felt hopeless; F3 = father felt restless/fidgety; F4 = father felt everything an effort; F5 = father felt worthless; F6 = father felt nervous; stratum = social stratum; space = age spacing between siblings; sexcon = sex constellation of siblings; edu_m = educational level of mother; edu_f = educational level of father; numchild = number of child within the family. The red line indicates the sample values, and the gray area shows the bootstrapped CIs. Mean bootstrap estimates, shown as black lines, are used to order the edges. Horizontal lines represent network edges, ordered from highest to lowest weight. Y-axis labels are omitted for clarity.

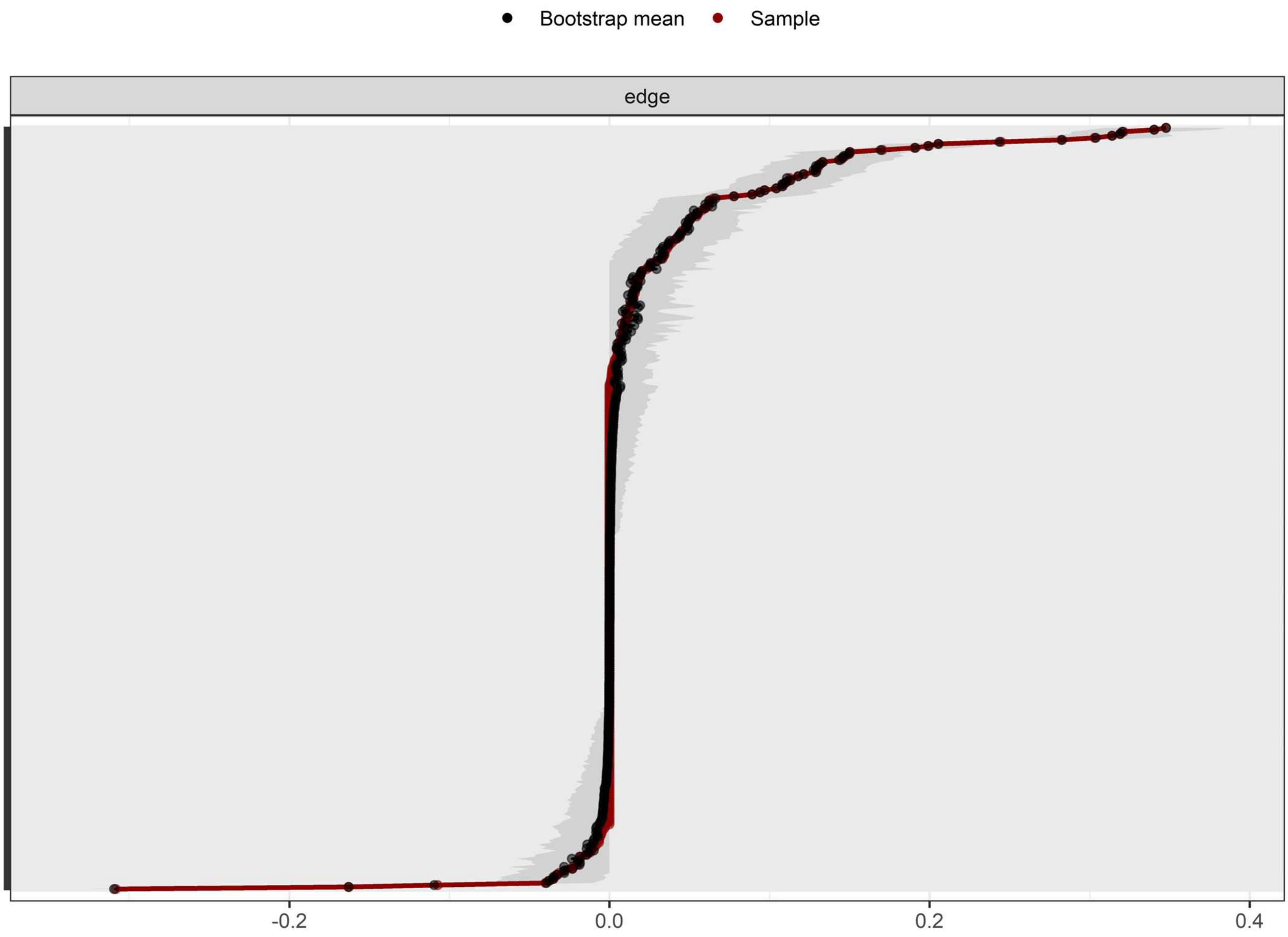


**Figure S13.** Bootstrapped 95% confidence intervals around each edge weight in the contemporaneous network at T2. YS = younger sibling; OS = older sibling; ES = emotion symptom; CP = conduct problem; H = hyperactivity; PP = peer problem; PS = pro-social; M1 = mother felt depressed; M2 = mother felt hopeless; M3 = mother felt restless/fidgety; M4 = mother felt everything an effort; M5 = mother felt worthless; M6 = mother felt nervous; F1 = father felt depressed; F2 = father felt hopeless; F3 = father felt restless/fidgety; F4 = father felt everything an effort; F5 = father felt worthless; F6 = father felt nervous; stratum = social stratum; space = age spacing between siblings; sexcon = sex constellation of siblings; edu_m = educational level of mother; edu_f = educational level of father; numchild = number of child within the family. The red line indicates the sample values, and the gray area shows the bootstrapped CIs. Mean bootstrap estimates, shown as black lines, are used to order the edges. Horizontal lines represent network edges, ordered from highest to lowest weight. Y-axis labels are omitted for clarity.

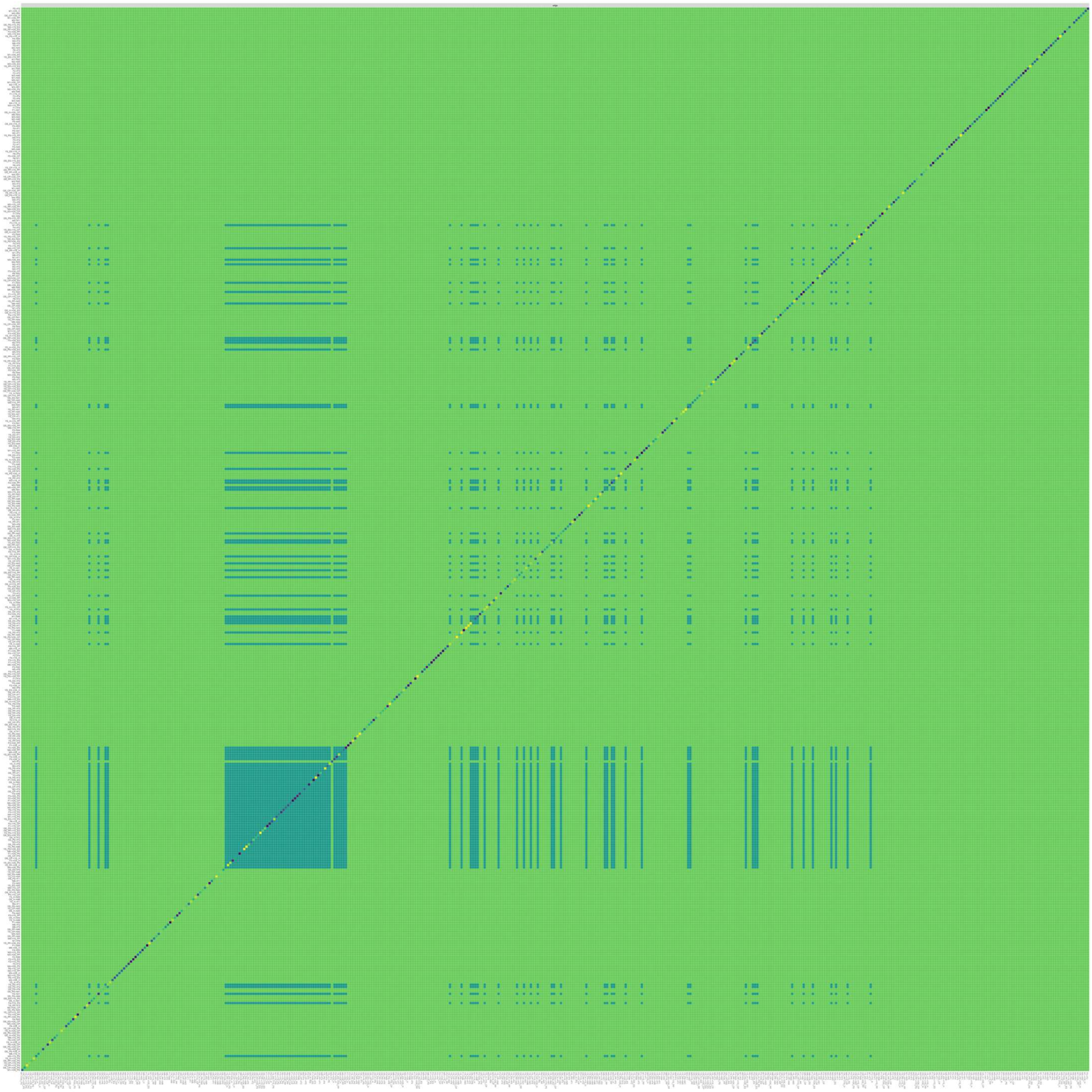

**Figure S14.** Bootstrapped difference tests of edges in the temporal network. YS = younger sibling; OS = older sibling; ES = emotion symptom; CP = conduct problem; H = hyperactivity; PP = peer problem; PS = pro-social; M1 = mother felt depressed; M2 = mother felt hopeless; M3 = mother felt restless/fidgety; M4 = mother felt everything an effort; M5 = mother felt worthless; M6 = mother felt nervous; F1 = father felt depressed; F2 = father felt hopeless; F3 = father felt restless/fidgety; F4 = father felt everything an effort; F5 = father felt worthless; F6 = father felt nervous. Blue boxes indicate edges that do not differ significantly from one another and green boxes represent edges that do differ significantly from one another. The color of the boxes on the diagonal corresponds to the edge values, with darker colors representing higher edge values and lighter colors representing lower edge values.

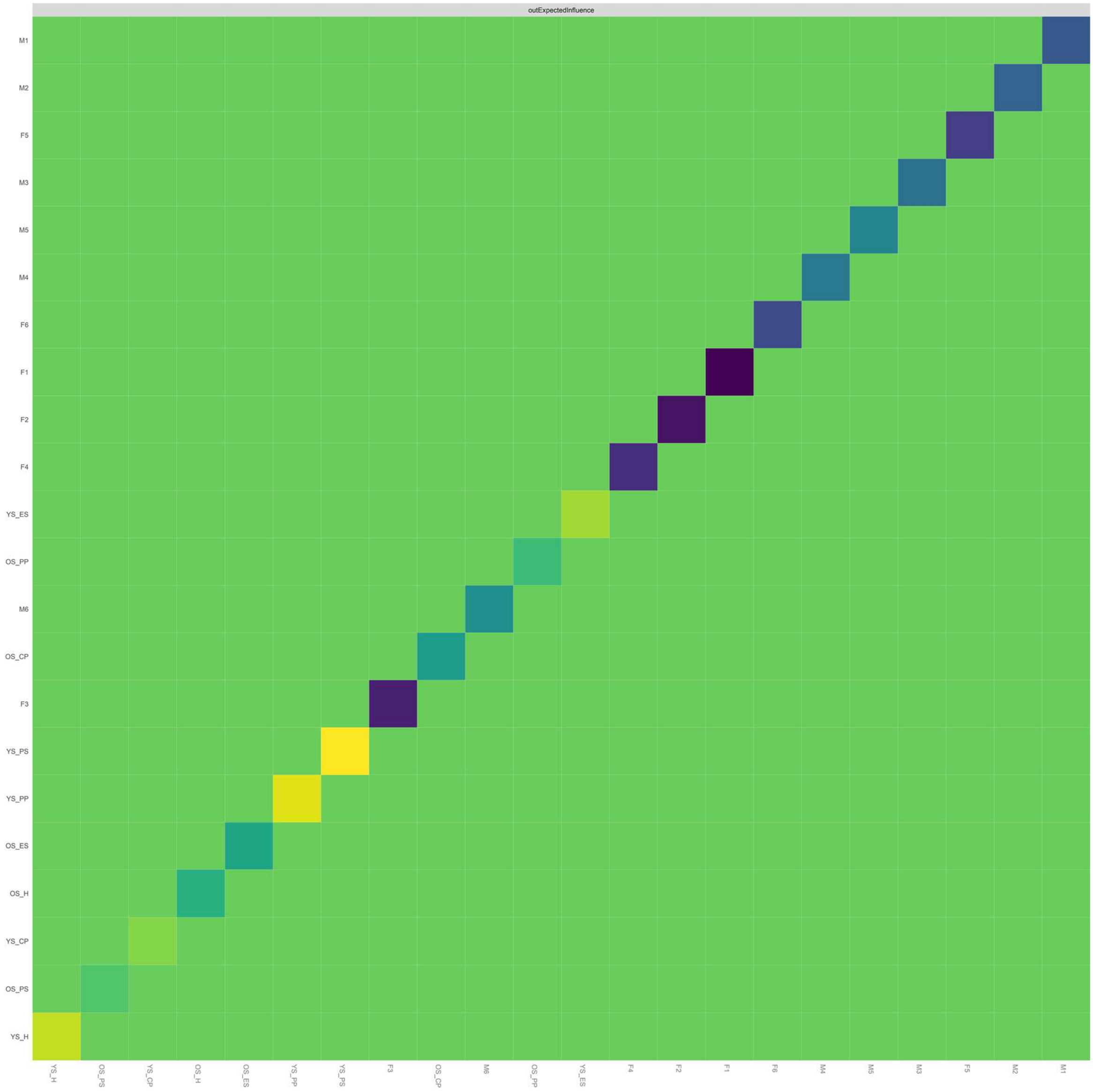


**Figure S15.** Bootstrapped difference tests of out-EIs in the temporal network. YS = younger sibling; OS = older sibling; ES = emotion symptom; CP = conduct problem; H = hyperactivity; PP = peer problem; PS = pro-social; M1 = mother felt depressed; M2 = mother felt hopeless; M3 = mother felt restless/fidgety; M4 = mother felt everything an effort; M5 = mother felt worthless; M6 = mother felt nervous; F1 = father felt depressed; F2 = father felt hopeless; F3 = father felt restless/fidgety; F4 = father felt everything an effort; F5 = father felt worthless; F6 = father felt nervous. Blue boxes indicate nodes that do not differ significantly from one another and green boxes represent nodes that do differ significantly from one another. The color of the boxes on the diagonal corresponds to the value of node out-EIs, with darker colors representing higher out-EIs and lighter colors representing lower out-EIs.

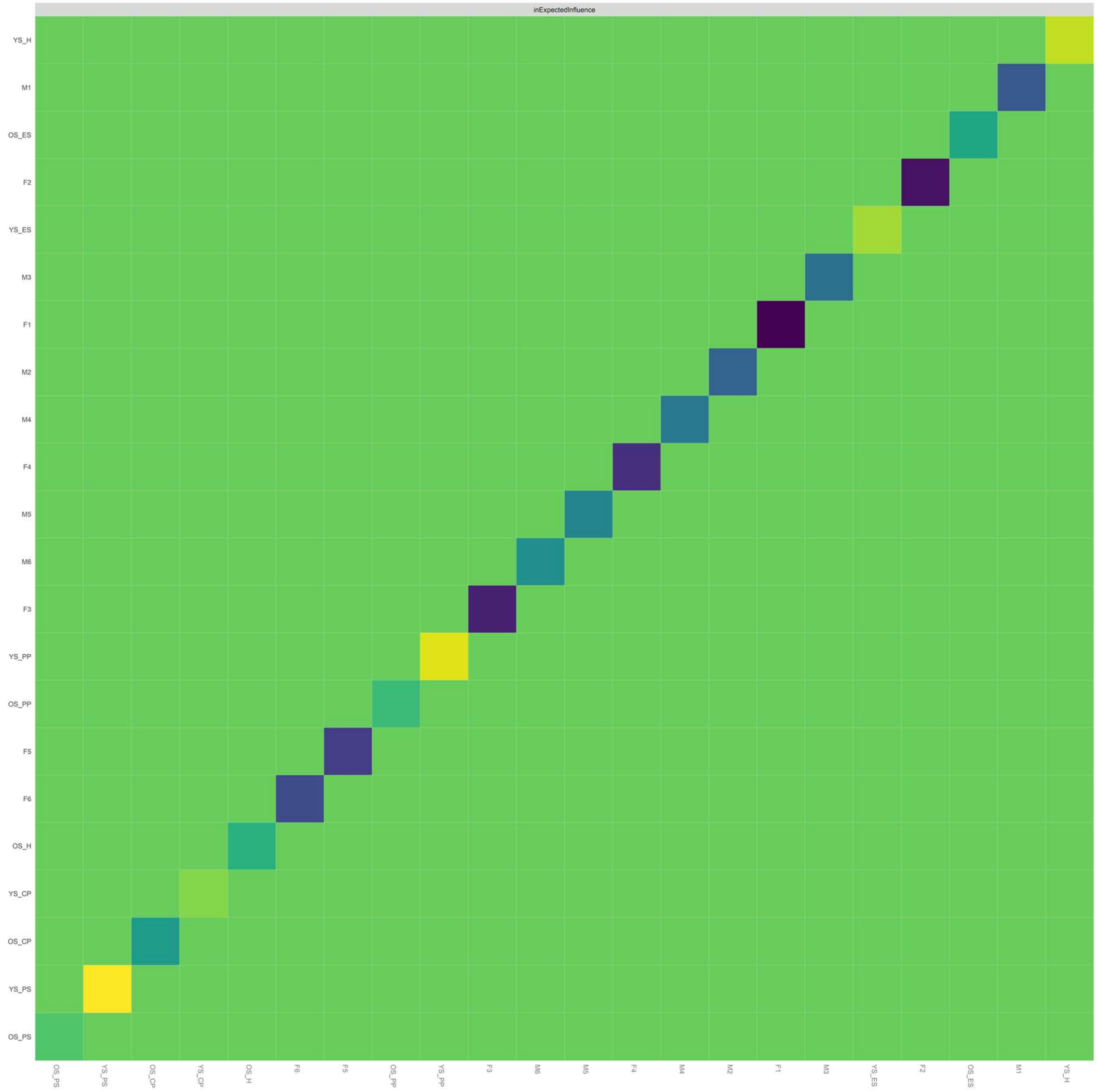


**Figure S16.** Bootstrapped difference tests of in-EIs in the temporal network. YS = younger sibling; OS = older sibling; ES = emotion symptom; CP = conduct problem; H = hyperactivity; PP = peer problem; PS = pro-social; M1 = mother felt depressed; M2 = mother felt hopeless; M3 = mother felt restless/fidgety; M4 = mother felt everything an effort; M5 = mother felt worthless; M6 = mother felt nervous; F1 = father felt depressed; F2 = father felt hopeless; F3 = father felt restless/fidgety; F4 = father felt everything an effort; F5 = father felt worthless; F6 = father felt nervous. Blue boxes indicate nodes that do not differ significantly from one another and green boxes represent nodes that do differ significantly from one another. The color of the boxes on the diagonal corresponds to the value of node in-EIs, with darker colors representing higher in-EIs and lighter colors representing lower in-EIs.

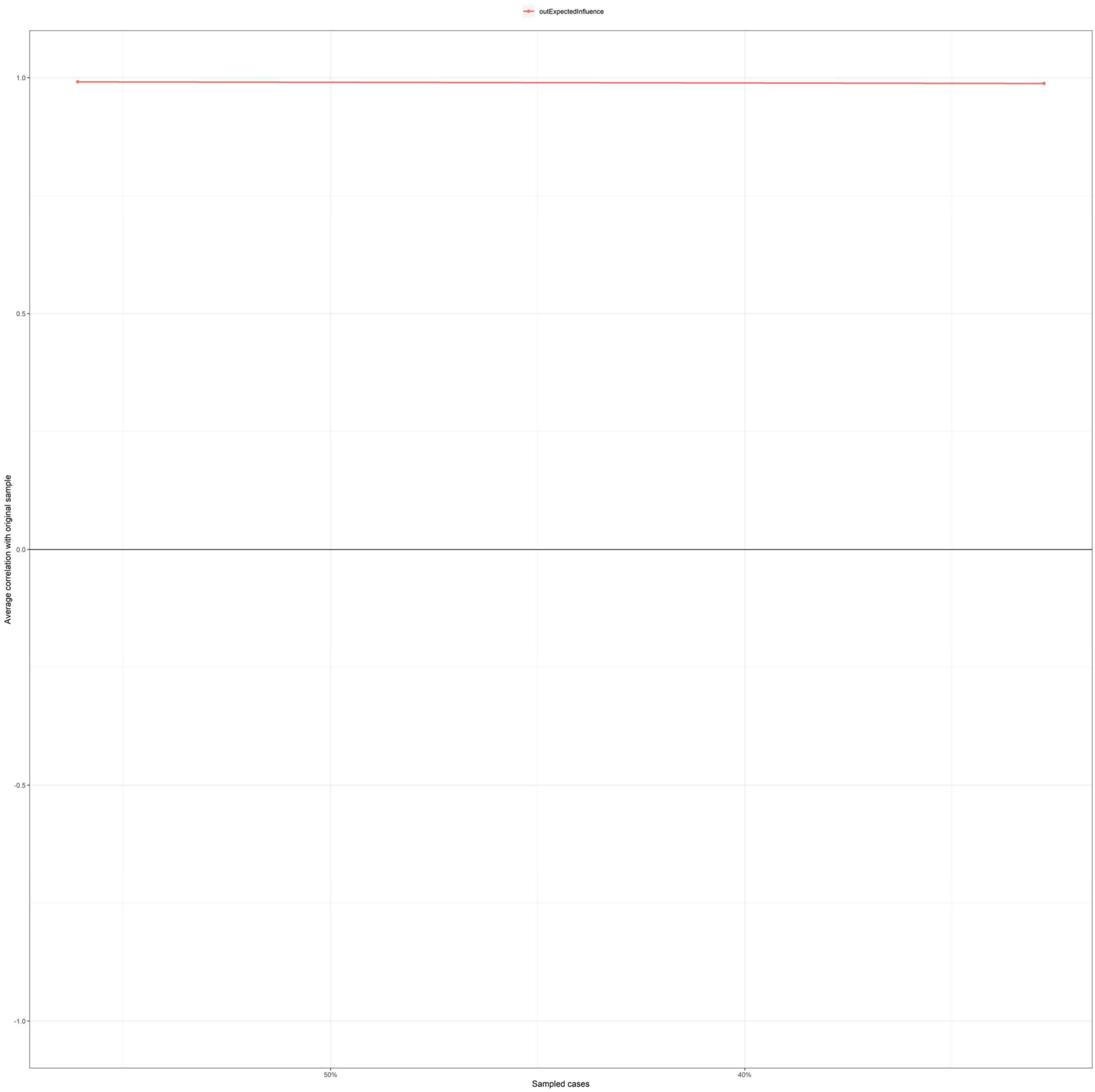


**Figure S17.** Stability of out-EI in the temporal network.

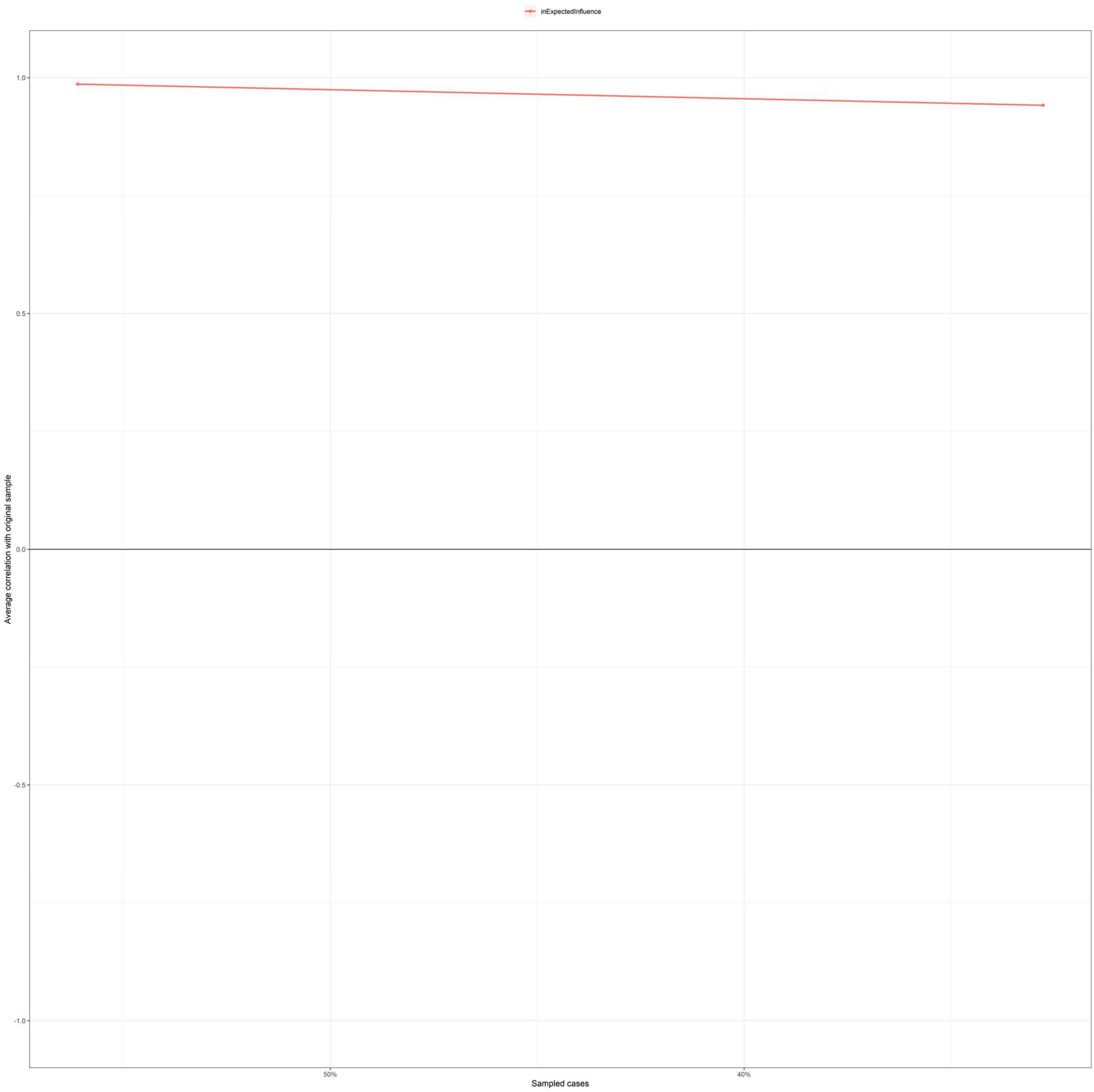


**Figure S18.** Stability of in-EI in the temporal network.

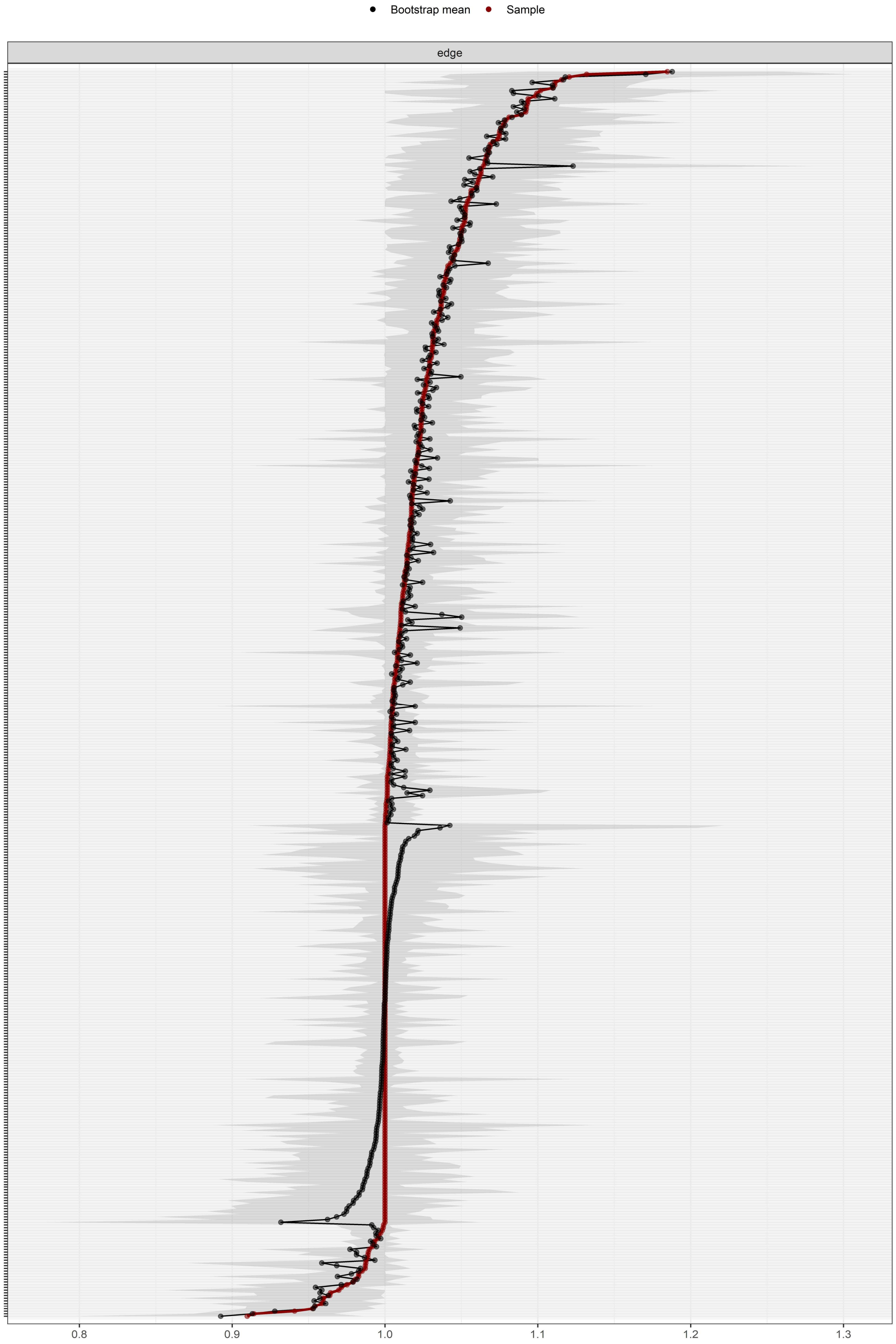


**Figure S19.** Bootstrapped 95% confidence intervals around each edge weight in the temporal network. The red line indicates the sample values, and the gray area shows the bootstrapped CIs. Mean bootstrap estimates, shown as black lines, are used to order the edges. Horizontal lines represent network edges, ordered from highest to lowest weight. Y-axis labels are omitted for clarity.
